# Comparative biodegradation analysis of three compostable polyesters by a marine microbial community

**DOI:** 10.1128/aem.01060-23

**Published:** 2023-11-28

**Authors:** Ingrid E. Meyer Cifuentes, Julius Degenhardt, Meina Neumann-Schaal, Nico Jehmlich, David Kamanda Ngugi, Başak Öztürk

**Affiliations:** 1 Junior Research Group Microbial Biotechnology, Leibniz Institute DSMZ - German Collection of Microorganisms and Cell Cultures, Braunschweig, Germany; 2 Research Group Metabolomics, Leibniz Institute DSMZ - German Collection of Microorganisms and Cell Cultures, Braunschweig, Germany; 3 Department of Molecular Systems Biology, Helmholtz-Centre for Environmental Research - UFZ, Leipzig, Germany; 4 Department of Microorganisms, Leibniz Institute DSMZ - German Collection of Microorganisms and Cell Cultures, Braunschweig, Germany; Shanghai Jiao Tong University, Shanghai, China

**Keywords:** plastic, marine microbiology, biodegradation, hydrolase

## Abstract

Biodegradable plastics are one of the possible solutions for reducing plastic waste, yet the mechanisms and organisms involved in their degradation in the aquatic environment remain understudied. In this study, we have enriched a microbial community from North Sea water and sediment, capable of growing on the polyester poly(butylene succinate). This culture was grown on two other biodegradable polyesters, polycaprolactone and a poly(butylene adipate-co-terephthalate) blend. The differences between microbial community structure and biodegradation activity on these three polymers were determined by metagenomics, metaproteomics, and respirometry analysis. In this study, we observed that the polymer type drives the community structure and determines its biodegradation capability. When the PBS-enriched culture grew on the poly(butylene adipate-*co*-terephthalate) blend, the community was more diverse, yet showed the lowest biodegradation percentage, while poly(butylene succinate) and polycaprolactone resulted in a less diverse community but much higher biodegradation activity. The dominating species were *Alcanivorax* sp., *Thalassobius* sp., or *Pseudomonas* sp., depending on the substrate. In general, we have observed that Gammaproteobacteria were more abundant and active within the biofilm on the polymers and Alphaproteobacteria within the free-living fraction of the enrichments. Three putative hydrolases were recombinantly expressed and their hydrolytic activity on all polymers tested was verified. In conclusion, we showed that all three plastics can be biodegraded by bacteria naturally occurring within the marine environment.

**IMPORTANCE:**

Biodegradable plastics can be used in applications where the end product cannot be efficiently recycled due to high levels of contaminations, e.g., food or soil. Some of these plastics have a dedicated end of life, such as composting, but their degradation in the marine environment is poorly understood. In this study we showed that marine microbial communities can degrade a range of biodegradable polymers with different physical and chemical properties and use these as a sole carbon source for growth. We have also provided insights into the degradation mechanisms using a combined metagenomic and metaproteomic approach. In addition, we have identified three new enzymes that are capable of degrading both aliphatic polymers and aliphatic-aromatic copolymers, which can be used for biotechnological applications.

## INTRODUCTION

Biodegradable plastics are manufactured as an alternative to conventional plastics for specific applications in order to reduce the environmental impact of plastics (1, 2). They usually contain ester linkages that are more amenable to microbial degradation than carbon-carbon backbones present in recalcitrant plastics such as polyethylene and polystyrene (3, 4). Aliphatic polyesters such as poly(butylene succinate) (PBS), polycaprolactone (PCL), polylactic acid, and aliphatic-aromatic polyesters such as poly(butylene-adipate-co-terephthalate) (PBAT) and its derivatives are examples of biodegradable plastics. These are used in specific applications where the end product is short-lived, has a high risk of loss or wear during usage, and/or where recycling is not possible or practical due to food or soil contamination.

Of these polymers, PBS is of special interest due to its mechanical properties which are similar to those of polyolefins (2, 4, 5) while also having the possibility of being manufactured as a bio-based polymer. Both building blocks, succinic acid (Su) and 1,4-butanediol (B), can be obtained from the fermentation of sugars or other biomasses such as lignocellulose (6–9). This polymer is certified compostable under industrial and home-composting conditions. To improve its mechanical and thermal properties, PBS can be blended with other polyesters, such as PLA (1, 10, 11) or PCL (12, 13). PBS has been certified as compostable in industrial composters and for home composting (https://www.mcpp-global.com/en/mcpp-asia/products/brand/biopbsTM/, last accessed 11.08.2023), but not as marine biodegradable.

PBS is degraded by several fungal isolates (14–17), mostly from the terrestrial environment. However, the biodegradation of PBS by bacterial isolates has not been frequently reported. A PBS-degrading *Bacillus* sp. was isolated from compost (18). In another study, several strains of aquatic and terrestrial bacteria which degraded poly(ethylene succinate) that belong to the order *Bacillales* were isolated, but these showed limited or no degradation of PBS. PBS exhibited poorer biodegradation behavior than other aliphatic polymers, such as PCL (19) and polyhydroxybutyrate (PHB) (20, 21). In a study comparing the microbial communities that colonize different types of plastics in seawater, PBS showed very little colonization by microorganisms compared to polyhydroxybutyrate-valerate and Mater-Bi (22).

Biodegradation of PBS in natural water has been assessed in recent studies, mostly under laboratory conditions. PBS blown films were completely degraded within 50–60 days in natural seawater and sand-containing tanks under laboratory conditions (23). In another study, PBS films showed negligible degradation in brackish water under *in vitro* conditions within 56 days (24). Other studies have also shown that the biodegradation of PBS in natural water samples is very slow (21, 25).

The enzymes that degrade PBS are predominantly cutinases (14, 26, 27). Cutinases are serine esterases that belong to the α/β hydrolase family (28). They degrade cutin, a waxy polyester on the surface of higher plants (28–30). Some cutinase-like enzymes can degrade synthetic polymers, including the aromatic polyester poly(ethylene terephthalate) (PET) (31–34). The substrate range depends on the enzyme. For example, some polyester-degrading esterases can degrade a range of different aliphatic polyesters, such as PBS, PLA, and PCL (35, 36). The PET-degrading enzyme (PETase) from *Ideonella sakaiensis* 201-F6 degrades a range of aromatic polymers, but cannot degrade aliphatic polymers (8). In contrast, Cutinase 1 from *Thermobifida cellulosilytica* can degrade both PET and aliphatic polymers, including PBS (7). In addition to cutinases, PBS and derivatives can also be degraded by some fungal (37, 38) and bacterial (39) lipases.

In this study, we have enriched a marine microbial community that is capable of using PBS as the only carbon source. In addition to PBS, the enriched culture was grown on the films of PCL, which was shown to degrade in the marine environment (21, 25) and ecovio FT, a PBAT-based blended biodegradable plastic which was shown to be degraded by a marine microbial consortium (40). The culture was capable of degrading all plastics tested. Analyzing the biofilm community on the plastic and the free-living community in the culture supernatant with metagenomics and metaproteomics separately, we determined the microbial community composition and the differences between communities growing on different plastics. We discovered genes/enzymes that potentially play a role in the degradation of the plastics investigated in this study. In addition, we have recombinantly expressed and verified the functions of three plastic degrading enzymes using HPLC-DAD and HPLC-MS. This study let us identify the role of polymer type in shaping the microbial community degrading plastics and identify the enzymes involved in the degradation.

## RESULTS

### Degradation activity of the enriched community on ecovio FT, PBS, and PCL

**Fig 1:**
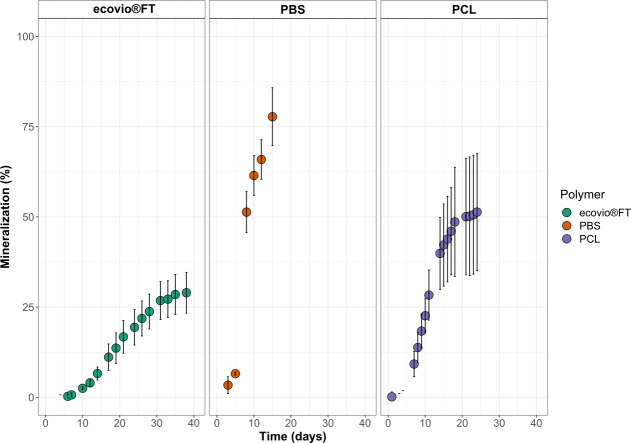
Mineralization and CO_2_ formation of ecovio FT, PBS, and PCL by the microbial community. Each measurement was made in three replicates. Error bars depict standard deviation. No CO_2_ formation was observed when polymers were incubated without the enriched culture.

The ability of the enriched culture to use ecovio FT, PBS, or PCL plastics as sole carbon sources was tested through mineralization tests. We observed that the culture was especially active when PBS or PCL was supplied as the carbon source. The enriched culture could convert 77.8 ± 8% of the initial carbon content supplied in the PBS films into CO_2_ (Fig. 1) already after 15 days of incubation. In contrast, the enriched culture converted less carbon to CO_2_ when PCL was supplied as the carbon source (51.4 ± 16.3% after 24 days of incubation). When PBS was supplied as the carbon source, the enriched culture could already convert 50% of the initial carbon content after 8 days of incubation. This indicates that PBS is degraded more rapidly than PCL by the enriched culture.

The enriched culture was able to mineralize less of the ecovio FT than PBS and PCL. It could convert only about 29 ± 5.7% of the initial carbon content into CO_2_, which took 33 days.

### The characterization of the enriched culture community

The metagenomes of the community growing on ecovio FT, PBS, or PCL were assembled, binned, and taxonomically classified. The metagenome binning resulted in 35 metagenome-assembled genomes (MAGs) of medium- (>75% completeness; *n* = 6) to high-quality (>95% completeness; *n* = 29 bins) with <3.5% contamination (Table S1). The most representative class of bacteria within the metagenomes were Alphaproteobacteria with 54.3% and Gammaproteobacteria with 14.3% of the total community. Other less common bacteria were Bacteroidia (8.6%) and Actinomycetia (5.6%). At the genus level, the most representative was *Sulfitobacter* sp. (PC bin 7, bin 9, and bin 38), followed by *Marinobacter* sp. (bin 43 and bin 48), *Thalassobius* sp. (PB bin 16 and bin 47), and *Amylibacter* sp. (bin 29 and bin 43). The complete breakdown of the taxonomic distribution of the community can be found in Fig. S1 through S5.

Differential gene abundance was determined by calculating log2 fold changes of genes found in the plastic film-attached fraction in comparison to the bacteria in the suspension (free-living). Additionally, MAGs were screened for enzymes that could potentially be involved in plastic degradation. Within this group, we identified 55 esterases, α/β-hydrolases, PETase-like enzymes (Ple), and lipases in total. Within the microbial community, *Alcanivorax* sp. (bin 24) and *Pseudomonas* sp. (bin 31) synthesized the highest number of enzymes of interest.

**Fig 2:**
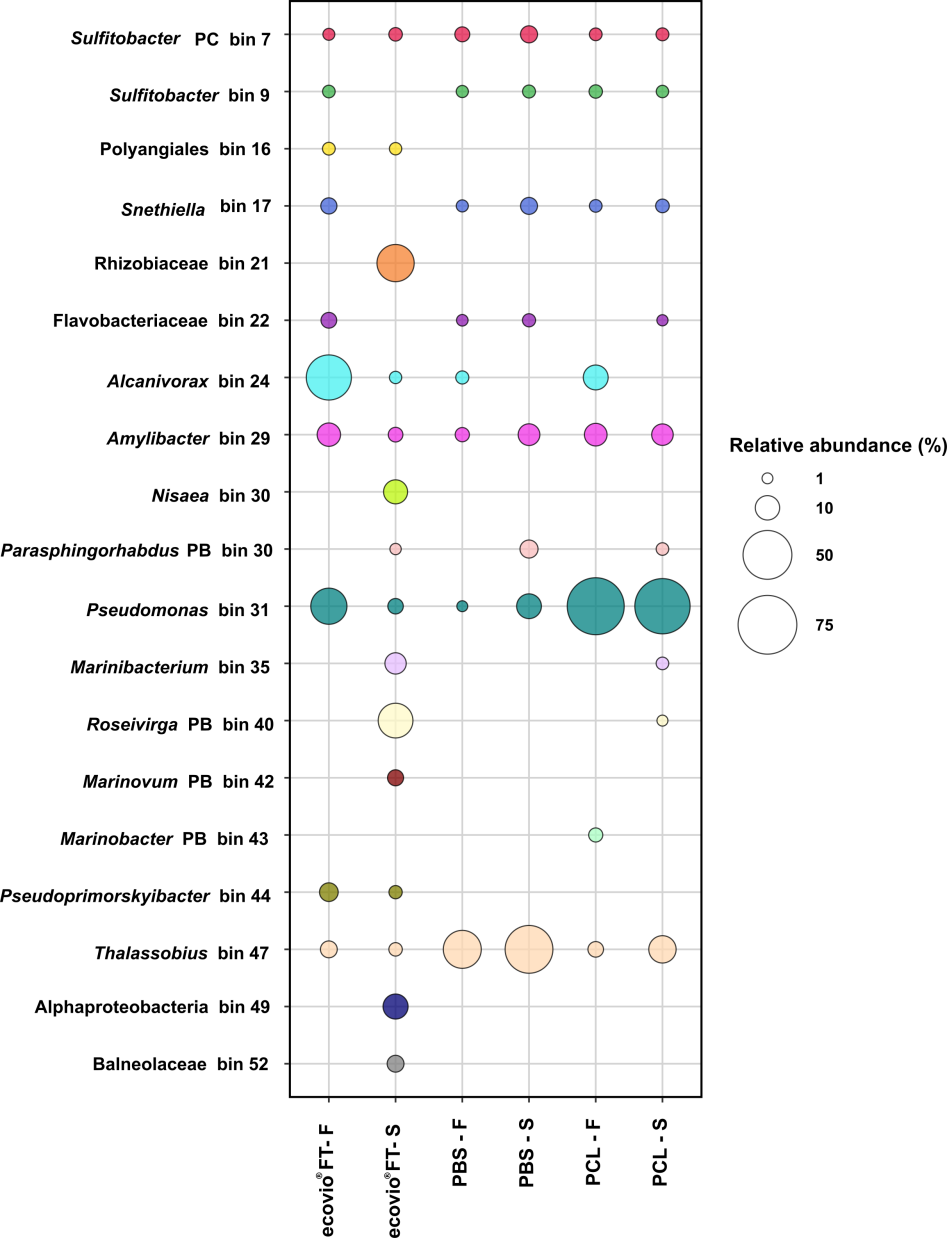
MAG abundance. Taxonomic abundance profile in the presence of ecovio FT, PBS, and PCL in the film-attached (F) and free-living fraction (S) is shown. The sizes of the bubbles represent the relative abundance (%) of each bin in the microbial community. The color of each bubble represents a taxon.

The relative abundance of each MAG was calculated for the different plastics tested. Among the film-attached bacteria that grew on PBS or PCL, the most abundant species was *Pseudomonas* sp. (bin 31) with 59.4% and 70.2% relative abundance, respectively (Fig. 2). On the ecovio FT film, the most abundant MAG was an *Alcanivorax* sp. (bin 24; 42% relative abundance), followed by *Pseudomonas* sp. (bin 31; 25.5% relative abundance). Interestingly, the abundance of *Alcanivorax* sp. (bin 24) on PBS (1.8% relative abundance) decreased almost sixfold compared to PCL (10.5%), where it was the second most abundant species. In the free-living fraction of PBS, the most abundant bacterium was *Thalassobius* sp. (bin 47) with 47.7% relative abundance. In the cultures amended with ecovio FT, two species belonging to the Rhizobiaceae family (bin 21) and *Roseivirga* sp. (PB bin 40) were highly enriched in the free-living fraction (27.2 and 23.2% relative abundance, respectively). These two bacteria (bin 21 and PB bin 40) were unique to the free-living fractions of ecovio FT cultures, and therefore not detected in the biofilms. The most abundant bacterium in the free-living fractions of the culture growing on PCL was also *Pseudomonas* sp. (bin 31), and its relative abundance was similar to that of the attached-living fraction (65.71%). In addition, two species of *Sulfitobacter* sp. (PC bin 7 and bin 9), *Snethiella* sp. (bin 17), and *Amylibacter* sp (bin 29) were present in similar relative abundance in all but one culture. *Pseudomonas* sp. (bin 31) and *Thalassobius* sp. (bin 47) were also ubiquitous and had the highest abundance when the culture grew on PBS. Apart from the aforementioned Rhizobiaceae (bin 21), six other species were detected exclusively when the enriched culture grew on ecovio FT, four of which were present only in the free-living fraction. The only other plastic-specific species detected was *Marinobacter* sp. (PB bin 43) in the biofilm on PCL.

### Bacterial activity based on protein synthesis

**Fig 3:**
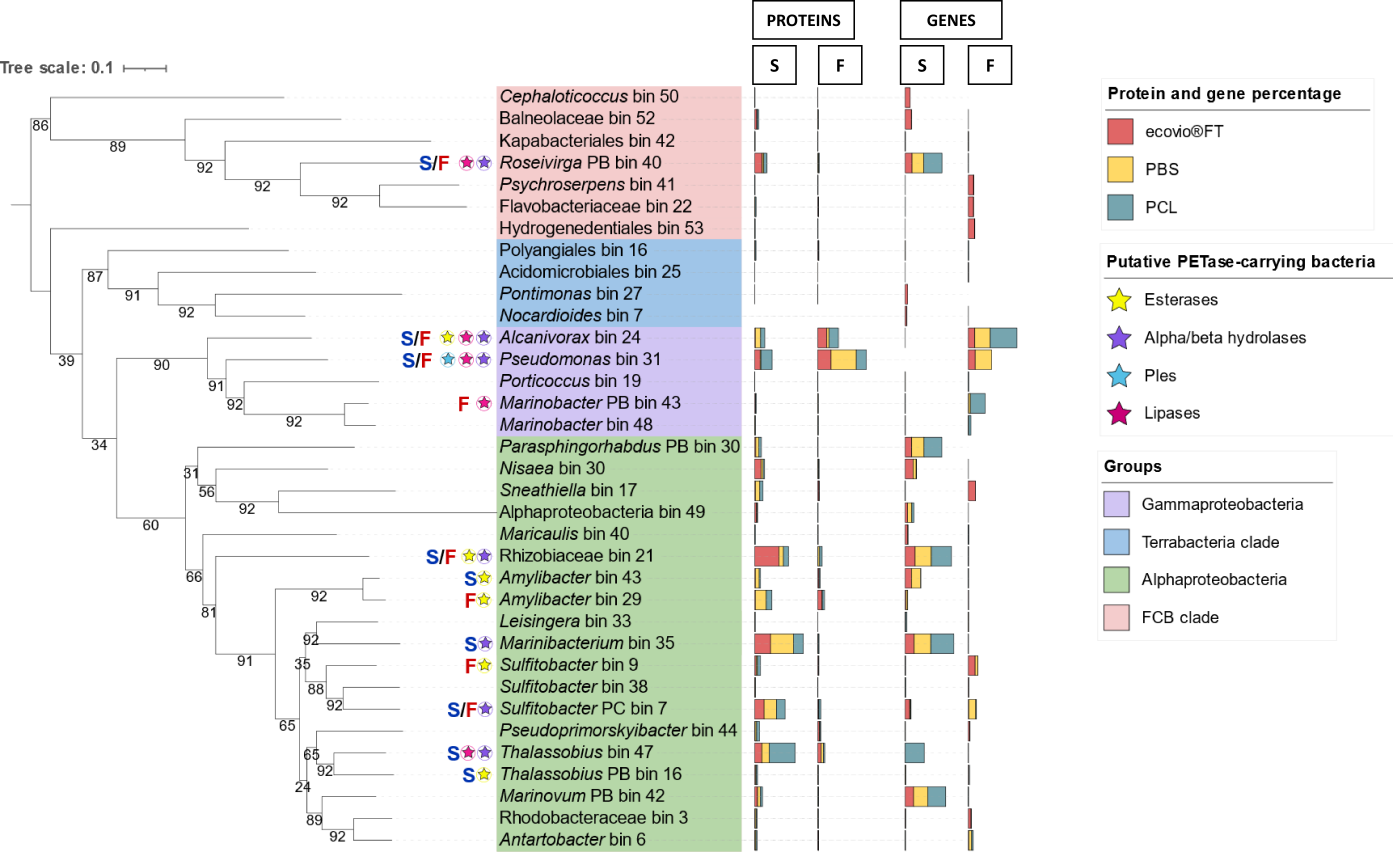
Gene/protein contribution of the MAGS and their phylogenetic relationship. The 35 MAGs identified in this study and the corresponding taxa are shown. Multibars on the right side of each taxa/MAG represent percentages of either genes or proteins that were significantly (≥0.05) found in the film-attached fraction (F) or in the free-living fraction (S) in ecovio FT (light red), PBS (light yellow), and PCL (dark cyan) cultures. A star on the left side of a taxa/bin, indicates the presence of an α/β hydrolase (purple), esterase (yellow), lipase (magenta), or Ples (light blue) found in a bin and that was significantly found in the film-attached fraction (shown as “F” in red), free-living fraction (shown as “S” in blue) or both fractions (shown as “S/F” in blue and red, respectively). MAGs are additionally clustered and highlighted by the following groups: Gammaproteobacteria (soft purple), Alphaproteobacteria (soft blue), Terrabacteria (soft blue) clade, and FCB clade (soft pink). The numbers on the branches represent bootstrap values. The phylogenetic tree was created by using the UBCG pipeline and default parameters (https://www.ezbiocloud.net/tools/ubcg) and rooted with a midpoint root.

After analyzing protein formation on the film-attached bacteria supplied with one of the tested plastics, we observed that most proteins were synthesized by the two species, *Pseudomonas* sp. (bin 31) and *Alcanivorax* sp. (bin 24). Surprisingly, PBS seems to trigger the activity of *Pseudomonas* sp. (bin 31), as more than half of the total synthesized proteins (~63%) (Fig. 3) were released by this species. Compared to *Pseudomonas* sp. (bin 31), the rest of the community was less active (<6% of synthesized proteins). When the enriched culture was supplied with ecovio FT or PCL, the activity of *Pseudomonas* sp. (bin 31) and *Alcanivorax* sp. (bin 24) was almost equal. In the presence of ecovio FT, *Pseudomonas* sp. (bin 31) produced 32.6% of the proteins and *Alcanivorax* sp. (bin 24) produced 22.3%. In the presence of PCL, 25.3% and 23.1% of the proteins were produced by *Pseudomonas* sp. (bin 31) and *Alcanivorax* sp. (bin 24), respectively. Other biofilm-active bacteria included a species of *Thalassobius* sp. (bin 47) and *Amylibacter* sp. (bin 29). They produced between 5% and 10% of proteins under different conditions, although *Thalassobius* sp. (bin 47) was less active in the presence of PCL (3.36% proteins formed). Conversely, *Thalassobius* sp. (bin 47) was the most active bacterium in the free-living fractions (22.21% proteins formed).

In the free-living fractions, the soluble degradation products of ecovio FT degradation mainly stimulated the activity of a Rhizobiaceae species (bin 21; ~21% of proteins formed) and some other bacteria such as *Marinibacterium* sp. (bin 35; ~14% of proteins formed). *Sulfitobacter* sp. (PC bin 7), *Roseivirga* sp. (PB bin 40), *Thalassobious* sp. (bin 47), and *Nisaea* sp. (bin 30) were also reasonably active by forming between 5% and 10% of synthesized proteins. *Pseudomonas* sp. (bin 31) showed some activity (4.61% of proteins formed), while *Alcanivorax* sp. did not appear to play a significant role in this fraction (<1% of proteins formed).

Similar to the effect observed when ecovio FT was supplied as a substrate, the PBS triggered the activity of *Marinibacterium* sp. (bin 35; ~20% of proteins formed), and it was the most active bacterium in the free-living fraction. Other active bacteria were *Sulfitobacter* sp. (PC bin 7), *Amilybacter* sp. (bin 29), and *Thalassobius* sp. (bin 47), contributing 5–10% of proteins formed. In this case, *Alcanivorax* sp. (bin 24) showed to have some activity (4.5% proteins formed) and *Pseudomonas* sp. (bin 31) was less active (~1% of proteins formed). Collectively, this suggests that *Pseudomonas* sp. (bin 31) may play an important role in biofilm formation, the degradation of PBS, or both.

### Synthesis of other proteins of interest

A total of 12,235 protein groups were detected in the metaproteomes of the cultures exposed to ecovio FT, PBS, and PCL films. Of these proteins, we detected the formation (at least two times) of 9,939 proteins in ecovio FT cultures (81.2%), 9,913 proteins in PBS cultures (81.0%), and 8,752 proteins (71.5%) in PCL cultures (Fig. S6). Among the three conditions, ecovio FT promoted higher differential expression of synthesized microbial proteins between the fraction adhered to the polymer film and the free-living fraction. Of the total amount of proteins produced in the presence of ecovio FT, the two fractions accounted for only 59%. In contrast, PBS and PCL shared ~72% of the proteins between the fraction adhered to the film and the free-living fraction.

In the film-attached and free-living fractions of the ecovio FT, PBS, and PCL-exposed communities, the most frequently synthesized proteins were transporters (ko02000), ribosomal proteins (ko03011), and proteins belonging to two-component systems (ko02022) (Fig. S7). Other proteins that were highly abundant were proteins involved in oxidative phosphorylation (ko00190), purine metabolism (ko00230), the citrate cycle (ko00020), and glycolysis (ko00010). We also detected additional metabolic pathways and proteins of interest that were less abundant but clearly produced both in the film-attached or free-living fraction. For example, proteins involved in secretion systems (ko02044) and biofilm formation in *Pseudomonas aeruginosa* (ko02025) were produced mainly in the film-attached fractions of the enriched cultures amended with ecovio FT and PBS (Fig. S7). The production of several proteins required for quorum sensing (ko02024) increased in free-living fractions of cultures supplied with PCL.

### Identification and abundance of Ples

Functional analysis of the metagenomes of ecovio FT, PBS, and PCL-exposed enrichment culture revealed several cutinases, lipases, esterases, and α/β-hydrolases potentially involved in the degradation of the plastic films. From these candidate genes, we selected 55 genes (Table S3) based on their protein synthesis (≥1 or ≤−1 of fold-change for upregulated or downregulated proteins, respectively) and significance (*P* value ≤ 0.05) either in the free-living or film-attached fractions of the enriched culture growing with ecovio FT, PBS, or PCL films. Three sequences of the most upregulated proteins that were also common to cultures growing with the different plastic films were retrieved and codon optimized for recombinant expression. These genes were named “*ple-tan1*,*”* “*ple-tan2*,” and “*ple-solo*.”

**Fig 4:**
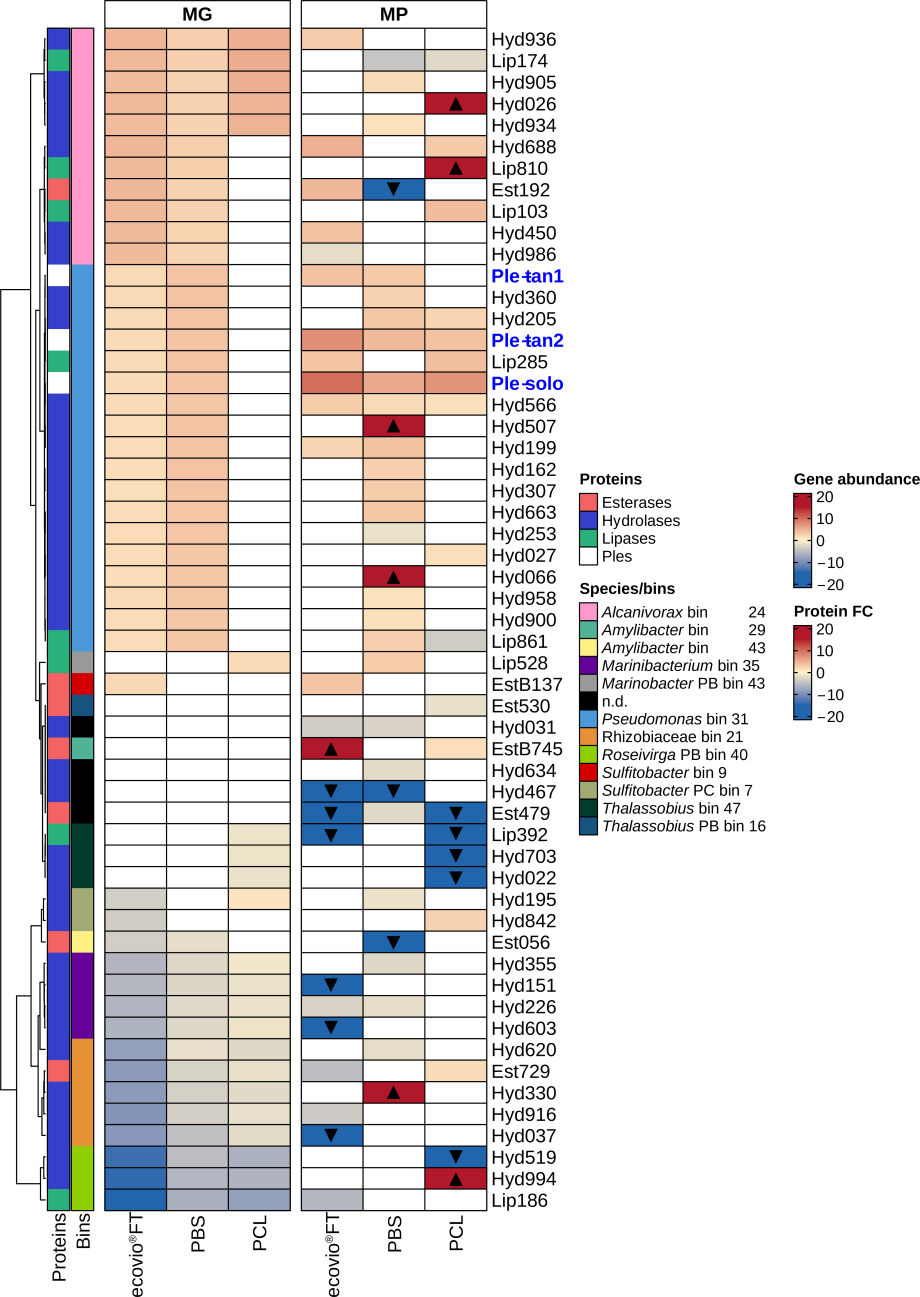
Gene relative abundance and fold change profiles of candidates of PETase-like genes detected in enriched cultures. We show α/β-hydrolases (blue), lipases (light green), esterases (soft pink), and Ples (white) as PETase-like candidate genes. Gene abundance and protein log2 fold changes were calculated from the metagenomes (MGs) and metaproteomes (MPs) of the microbial community growing with either ecovio FT, PBS, or PCL. Only genes or proteins with significant (≤0.05) and high (≥1 or ≤−1) fold changes are included. Blank cells indicate genes or proteins that were either not detected or not-significantly formed. The average of at least two biological replicates was used to calculate fold changes. Black-filled triangles indicate proteins unique in the film-attached fraction (up-pointing triangle) and unique in the free-living fraction (down-pointing triangle). The scale for gene and protein abundance shows the log2 fold changes. Abundant genes and proteins in the film-attached fraction are shown in shades of red. Abundant genes and proteins in the free-living fraction are shown in shades of blue. Taxa are represented by different colors. Samples were clustered according to the highest abundance in the metagenome.

The *ple-tan1, ple-tan2*, and *ple-solo* genes were more abundant in the metagenomes of the biofilm on ecovio FT and PBS than in the metagenomes of the PCL biofilm. Also, the protein Ple-tan1 was detected only on ecovio FT and PBS films, while both Ple-tan2 and Ple-solo formed in the film-attached communities of all polymers. However, the fold changes of protein formation were higher on the ecovio FT film than on the other plastics (Fig. 4).

Other genes associated with PETases were more abundant in enriched cultures containing ecovio FT and PBS (Fig. 4). When comparing fold changes of individual genes between the attached and free-living fractions, genes from *Alcanivorax* sp. (bin 24) showed a higher fold change in gene abundance in the biofilm of ecovio FT than on PBS, with a reverse trend for genes belonging to *Pseudomonas* sp. (bin 31). In addition, the esterase EstB137 from *Sulfitobacter* sp. (bin 9) was exclusively detected in the ecovio FT enriched cultures. In the presence of PCL, four hydrolases and one lipase belonging to *Alcanivorax* sp. (bin 24) were more abundant on the biofilm. Additionally, Lip528 of *Marinobacter* sp. (PB bin 43) and Hyd195 of *Sulfitobacter* sp. (PC bin 7) also had increased relative gene abundances. The production of the selected enzymes of interest was more heterogeneous compared to their gene abundances.

### Ple purification and identification

The genes *ple-tan1*, *ple-tan2*, and *ple-solo* were all present in the *Pseudomonas* sp. (bin 31) genome. The *ple-tan1* and *ple-tan2* genes were found to be in a tandem configuration with no other open reading frames in between, whereas *ple-solo* was identified on a separate contig. The Ple-tan1 and Ple-tan2 are phylogenetically related, while Ple-solo seems to belong to a different cluster (Fig. S8). These three enzymes are similar to several cutinases from *Marinobacter* and *Pseudomonas* species. Ple-tan1, Ple-tan2, and Ple-solo have 53%, 54%, and 48% amino acid identity, respectively, to the *Ideonella sakaiensis* PETase (A0A0K8P6T7, *Is*PETase). They are also closely related (61–66% amino acid identity) to the previously identified Ple628 and Ple629, which were identified from another marine consortium (40). In addition, we observed that Ple-tan1 and Ple-tan2 have an amino acid identity of 84.1%, while both have only 69% identity to Ple-solo.

To evaluate their activities, we successfully expressed the Ples recombinantly. The theoretical molecular weight of the Ples is 32.5 kD. The molecular weight of the expressed Ples corresponds to their theoretical molecular weight. The calculated yield after purification was 58.32, 18.56, and 33.28 mg for Ple-tan1, Ple-tan2, and Ple-solo in 1 L cell culture, respectively.

We investigated the temperature range in which the three Ples are most active by incubating them at different temperatures with ecovio FT as the substrate and measuring the formation of terephthalate-butanediol monoester (BT) and terephthalic acid (T). Ple-tan1, Ple-tan2, and Ple-solo had the highest amounts of the ecovio FT degradation products, BT + T, after 48 h of incubation. The highest amounts of BT + T were produced at 20°C for the three enzymes, and decreased significantly at 40°C and 50°C (Fig. S5). Ple-tan1 was the most active of the three Ples even when incubated at 40°C. At 20°C, Ple-tan1 formed 611.3 ± 34.2 µM of BT + T after 48 h of incubation and at 40°C, the amount of BT + T decreased by 17.5% (106.9 ± 9.8 µM of BT + T). In contrast, Ple-tan2 and Ple-solo were more sensitive to higher temperatures and produced only 21.5 ± 1.1 and 18.1 ± 0.5 µM of BT + T, respectively, at 40°C after 48 h of incubation. The shift from 40°C to 50°C did not negatively affect the activity of Ple-tan2 and Ple-solo any further, while the activity of Ple-tan1 decreased considerably to 32% (relative to the amount of BT + T formed at 40°C after 48 h).

### Michaelis-Menten of Ples with pNPB

Catalytic constant (*k*cat) and Michaelis-Menten constant (*Km*) values were calculated by measuring the activity of Ple-tan1, Ple-tan2, and Ple-solo (0.005 µg/µL; 0.15385 µM) on para-nitrophenyl butyrate (pNPB) at 20°C. To calculate the Michaelis-Menten parameters, we tested different concentrations of pNPB (from 100 to 2,400 µM). The highest *kcat* (386.19 s^−1^) was observed for Ple-tan2, followed by Ple-tan1 (142 s^−1^), while Ple-solo had the lowest *kcat* (0.12 s^−1^). The *Km* was estimated as 454 ± 54, 1417 ± 94, and 254 ± 55 µM for Ple-tan1, Ple-tan2, and Ple-solo, respectively.

### Ple activity and product formation after ecovio FT, PBS, and PCL degradation

**Fig 5:**
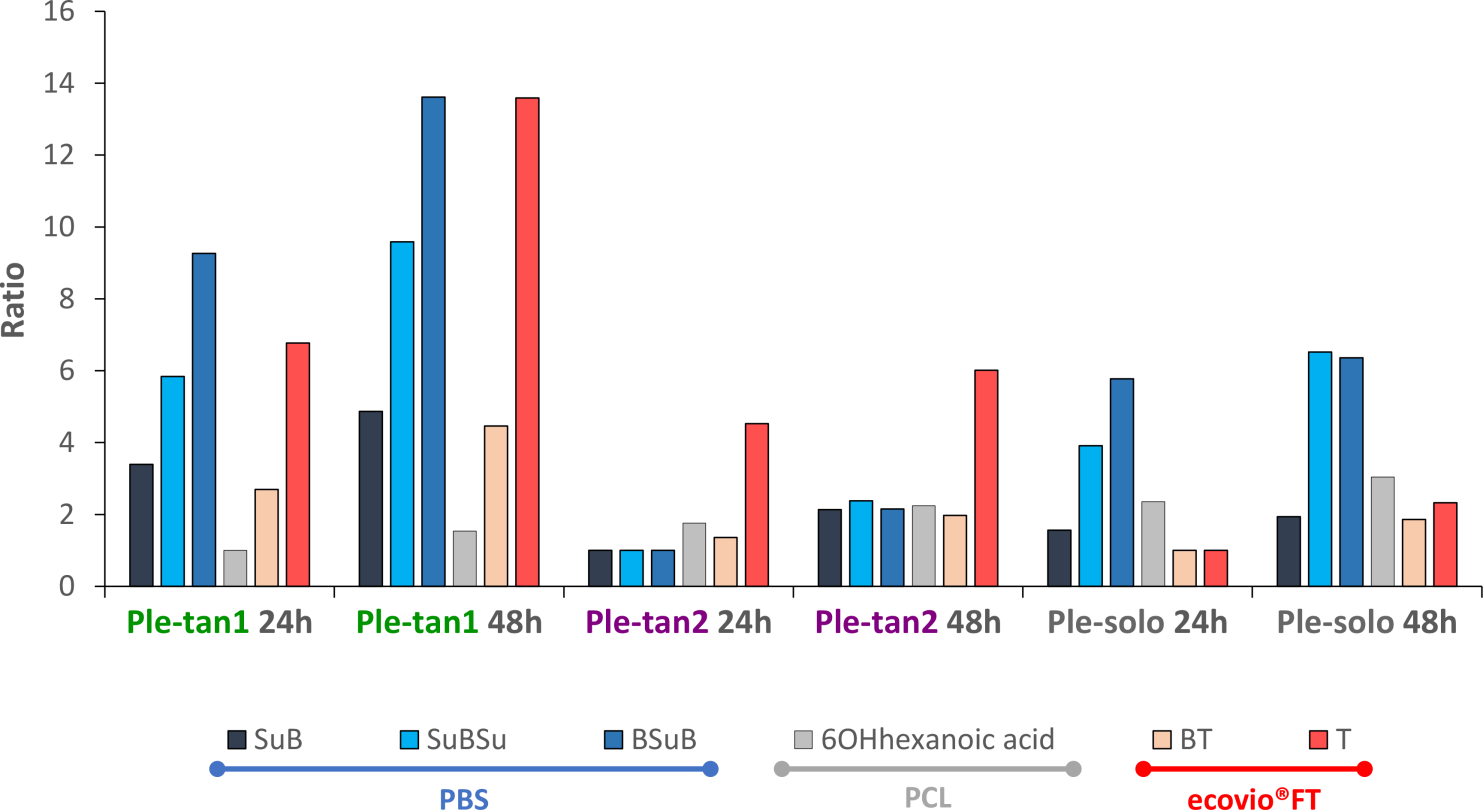
Hydrolysis of ecovio FT, PBS, and PCL by Ple-tan1, Ple-tan2, and Ple-solo. For each plastic type, the smallest peak area for each degradation product across all experiments was assigned as one. The ratios represent the fold change from the smallest peak area across all experiments. The products SuB (succinic acid + 1,4-butanediol), SuBSu (succinic acid + 1,4-butanediol + succinic acid) and BSuB (1,4-butanediol + succinic acid + 1,4-butanediol) were detected from PBS (blue line) growing cultures; 6OH hexanoic acid (6-hydroxyhexanoic acid) was detected from PCL (gray line) growing cultures and BT (terephthalate-butanediol monoester) and T (terephthalic acid) were detected from ecovio FT (red line) growing cultures. Each ratio was calculated using the average of three replicates.

Ple-tan1, Ple-tan2, and Ple-solo were able to hydrolyze all polymers tested. The ecovio FT was primarily degraded to BT. The T production was less than 10% of the total products in each case. After 48 h, Ple-tan2 and Ple-solo produced similar amounts of total products, while Ple-tan1 produced two times the amount of either enzyme (Fig. 5). Because the degradation products of PCL and PBS are not UV active, they were detected using LC-MS. The degradation products of PBS were oligomers of Su and B. After 48 h, Ple-tan1 produced the largest amount of the monoester SuB (succinic acid + 1,4-butanediol) and the diesters BSuB (1,4-butanediol + succinic acid + 1,4-butanediol) and SuBSu (succinic acid + 1,4-butanediol + succinic acid), followed by Ple-solo. The monomer Su was not detected due to rapid elution from the column. B was detected mainly in the samples treated with Ple-solo after 48 h. When PCL was used as substrate, Ple-solo again produced the largest amount of the monomer 6-hydroxyhexanoic acid (6OHhexanoic acid), followed by Ple-tan2 and Ple-tan1 (Fig. 5).

## DISCUSSION

### The effects of plastic type on microbial community composition and activity

Of all three plastics, PBS and PCL showed a high percentage of mineralization. The degradation of ecovio FT not only took longer under the tested conditions, but the overall conversion to CO_2_ was also lower. This is likely due to its terephthalate moieties, as the original culture was enriched on an aliphatic polyester, and we could not detect terephthalate degradation gene clusters in the metagenomes (Fig. S2). Thus, this community is able to partially degrade but not fully mineralize this polymer. The downstream degradation products of PBS and PCL, on the other hand, do not have the terephthalate moiety and are readily degradable by a wide range of microorganisms.

In each experiment, the type of plastic polymer added as a carbon source resulted in changes in the microbial community composition. This is best seen in the high number of MAGs detected exclusively when culturing with ecovio FT compared to experiments with PBS and PCL, especially within the free-living fraction. This is probably because a broader spectrum of degradation products is released during the degradation of ecovio FT compared to PBS and PCL, resulting in the enrichment of a greater diversity of microorganisms that can consume these products.

The biofilm communities on all plastic films were dominated by either *Alcanivorax* sp. (bin 24), *Pseudomonas* sp. (bin 31), or *Thalassobius* sp. (bin 47). *Alcanivorax* is well known for its ability to degrade aliphatic polyesters including PCL (20, 41, 42) and recently also was reported to degrade low-density polyethylene (41). Members of genus *Pseudomonas* have also been reported to degrade a wide range of plastic polymers including low-density polyethylene (43), high-density polyethylene (44), polystyrene (20), and polyester polyurethane (20, 41, 43–45). In this study, both strains carried genes encoding proteins that were either putative plastic-degrading enzymes, or enzymes that were proven in this study to degrade all three polyesters. In accordance with the abundance data, gammaproteobacterial members *Alcanivorax* sp. (bin 24) and *Pseudomonas* sp. (bin 31) had more upregulated proteins and were thus more active on the biofilm. It is likely that *Alcanivorax* sp. (bin 24) and *Pseudomonas* sp. (bin 31) are the primary plastic degraders of the consortium. The role of *Pseudomonas* sp. (bin 31) was further confirmed by the purification and characterization of the three novel Ples. Several other marine Gammaproteobacteria such as *Shewanella* sp. CT01, and *Moritella* spp. CT12 and JT01 degrading PCL (20) and a *Marinobacter* sp. from another ecovio FT-degrading marine enrichment (40, 46) were previously documented, demonstrating the capability of certain marine Gammaproteobacteria to degrade plastics.

The role of the alphaproteobacterial members of the community is less clear. MAGs such as *Amylibacter* sp. (bin 29), *Marinibacterium* sp. (bin 35), and *Rhizobiaceae* (bin 21) had more upregulated proteins in the free-living fractions. The observation that Alphaproteobacteria are more active in the free-living fractions of plastic degrading cultures was previously made for another marine consortium that grew on ecovio FT (40). We propose that these species consume the oligomeric or monomeric degradation products, which are released to the culture supernatant.

*Thalassobius* sp. (bin 47), the most abundant organism in the consortium when PBS was supplied as a substrate, has not yet been described to degrade plastic. However, it has been reported to degrade polycyclic aromatic hydrocarbons (47) and phthalate esters (48). Although this microorganism was abundant, we did not detect a clear difference in the protein abundance profiles of this microorganism between the biofilm and free-living fraction, showing that this organism is similarly active in both fractions. At the moment, the role of this microorganism in plastic degradation is unclear. The presence of α/β-hydrolases and esterases in the genome, in conjunction with the high abundance of this organism in the PBS biofilm, suggests that it could be actively degrading PBS. However, the high number of upregulated proteins, including the hydrolases and esterases, in the free-living fractions suggests that the organism is more specialized towards consuming the soluble degradation products.

### Characterization of the novel Ples

The activities of the characterized Ples on ecovio FT were lower than those of the previously identified marine PETase Ple629, but higher than that of Ple628 (46). The Ple with the highest activity, Ple-tan1, formed about 70% of the total degradation products of Ple629 after 48 h, while Ple-tan2 and Ple-solo formed 50% and 30%, respectively. Similarly, for the polymeric substrates, the difference between the Ple-tan1/Ple-tan2 tandem pair was not as drastic as between Ple628/Ple629, with Ple628 forming about 10-fold fewer degradation products than Ple629 (46). Although Ple-solo showed the lowest activity on p-NPB and ecovio FT, it had the highest activity on PCL, and the second-highest activity on PBS. The presence of several different esterases with different substrate ranges and efficiencies may give the microorganism the ability to degrade a broad range of polymers.

Although Ple-solo had the lowest activity on ecovio FT, it was more highly upregulated on this film compared to the other polymers. Cutinases and cutinase-like enzymes have been previously reported to be induced and expressed differentially within the same organism (46, 49, 50). Their expression can be constitutive or induced by the degradation products. It is possible that the degradation products of ecovio FT induce Ple-solo, despite its lower efficiency.

### Conclusions

Using a combination of cultivation, metagenomics, metaproteomics, and recombinant gene expression, we investigated the role of plastic type in shaping the composition of a marine-enriched culture that can degrade biodegradable plastic blends. Our findings show that plastic type influences microbial community composition, particularly in the free-living bacterial communities that consume the soluble degradation products. Our results also suggest that the marine ecosystem harbors microorganisms and enzymes capable of degrading a range of polyesters with different compositions and properties. This study provides evidence that *Alcanivorax* sp. (bin 24) and *Pseudomonas* sp. (bin 31) are relevant plastic degraders in marine environments, and the cutinases Ple-tan1, Ple-tan2, and Ple-solo have been shown to degrade PBS, PCL, and PBAT polymer blends.

## MATERIALS AND METHODS

### Sample collection

Seawater and coastal aerobic sediment for samples were collected from List/Sylt Germany (55°01′30.5″N 8°25′53.0″E) in June 2019. Samples were stored in the dark at 4°C until use.

### Reagents

The ecovio FT foil, a blend of PBAT and PBSeT (poly (butylene-sebacate–*co*–terephthalate)), was supplied by BASF (Ludwigshafen, Germany), PBS was supplied by PTT MCC Biochem, Thailand, and PCL was supplied by Futation (Denmark).

The ecovio FT, PBS, and PCL films were sterilized by submersion in 70% ethanol for 20 min. After this time, the plastic foils were removed with sterile tweezers and placed on open sterile Petri dishes to air dry aseptically.

### Culture conditions

All cultivations were carried out in a mineral medium M13a (DSM media no. 600a) with some modifications: Artificial seawater (ASW 1×), Hutner’s salts and metals solutions were prepared according to DSMZ 590 medium. The carbon and nitrogen sources described for these media (600a. M13a and 590 DSMZ media) were replaced by NH_4_Cl (1 mM) and K_2_HPO_4_ (0.1 mM).

The enrichment was performed as previously described (40). Briefly, 1 g of sediment was mixed with 10 mL of seawater and vortexed at top speed for 30 s to dislocate the cells from the sediment. After letting the mixture settle briefly, 1 mL of the supernatant was used to inoculate sterile 50 mL of mineral medium in a 250-mL flask, supplemented with a 5 × 5 cm^2^ PBS film, and 0.1% yeast extract. The cultures were incubated in a MaxQ 4000 orbital shaker (Fisher Scientific, Germany) at 22°C, 120 rpm. After disintegration of the film was observed (ca. 2 months), 1 mL of the culture was transferred into a flask with the same amount of fresh medium and PBS film as before, omitting the yeast extract. The procedure was repeated until a stably degrading culture was obtained.

### Mineralization assays

To measure mineralization of ecovio FT, PBS, and PCL, a pre-culture was prepared consisting of 1 mL pregrown enriched culture, supplied with a 5 × 5 cm^2^ PBS film. The pre-culture was grown with 50 mL of mineral media in a 300-mL Erlenmeyer flask and incubated at 20°C, 120 rpm in a MaxQ 4000 orbital shaker (Fisher Scientific, Germany). Pre-cultures were then diluted to 1:10 with mineral media and 1 mL of the dilution was inoculated in 25 mL of mineral media supplied with 1 mg carbon/L of either ecovio FT, PBS, or PCL. Each mineralization flask (250 mL volume) included an external CO_2_ trap containing 4 mL NaOH (0.1 mM). The purpose of the trap was to measure mineralization through the analysis of CO_2_ formation. Each condition consisted of three replicates inoculated with bacteria and supplied with either of the plastic films. Negative controls were prepared also in triplicate and consisted only of cultures without the carbon source. The cultures were incubated in a MaxQ 4000 orbital shaker (Fisher Scientific, Germany) at 22°C, 120 rpm. The tests were run until no further mineralization was detected. This was 15 days for PBS, 24 for PCL, and 39 for ecovio FT.

The entrapped CO_2_ was removed and diluted 10× with Milli-Q water in TOC glass vials. The carbon content was then quantified by using a calibration curve of NaHCO_3_. The CO_2_ formed in the mineralization assays was measured as inorganic carbon using a TOC-L analyzer and data collection was retrieved by using the TOC-Control L v.1.06 software (Shimadzu, Japan).

### DNA and protein extraction, purification, and quantification

For DNA and protein extraction, 1 mL of pre-culture was inoculated to 99 mL of marine mineral media supplied with 5 × 5 cm^2^ films of either ecovio FT, PBS, or PCL. Each condition consisted of four replicates. The cultures were incubated at 20°C, 120 rpm in a MaxQ 4000 orbital shaker (Fisher Scientific, Germany). Before film disintegration, the cultures were harvested by filtering the whole content of each flask through a 50 µM sieve, which was previously sterilized with ethanol. The free-living bacterial fraction of each culture was collected with clean beaker flasks placed under the sieve and transferred to clean 50 mL Falcon tubes. Each plastic film (attached bacterial fraction) retained on the sieve was washed 3× with Milli-Q water. Subsequently, the plastic films were separately transferred to sterile Petri dishes and submerged in 5 mL of phosphate buffer, pH 7.4. While submerged, biofilms were scratched out of each plastic film with clean scalpels. The suspended biofilms were homogenized by pipetting and transferred to sterile 50 mL Falcon tubes. Free-living and attached fractions were centrifuged at 10,000 rpm for 10 min at 4°C. Pellets were then stored at −80°C. Finally, DNA and proteins were extracted from both fractions simultaneously with the Quick-DNA/RNA Miniprep Plus Kit (Zymo Research, USA) according to the manufacturer’s instructions.

The integrity of the isolated DNA was analyzed in a 1.2% agarose gel and visualized with the Intas Gel v.0.2.14 software. Before sequencing, RNA contamination was removed from the DNA samples with RNase A (AppliChem, Germany). The quality of DNA was additionally analyzed by measuring the 260/280 and 260/230 ratios via Nanodrop by using the Nanodrop 2000 v.1.5 software (Nanodrop 2000, Thermo Fisher Scientific, USA).

The fractions of proteins isolated with Quick-DNA/RNA Miniprep Plus Kit (Zymo Research, USA) were further precipitated with acetone according to the manufacturer’s instructions. The concentration of the purified proteins as well as DNA were measured by using Qubit Protein Assay and Qubit dsDNA BR kits, respectively, and measured on a Qubit 3.0 Fluorometer (Invitrogen, USA).

### DNA sequencing

The DNA sequencing library was generated from 200 ng DNA using NEBNext Ultra II FS DNA Library Prep Kit for Illumina (New England BioLabs, USA) according to the manufacturer’s protocols including PCR Amplification with five cycles. The library was treated with Illumina Free Adapter Blocking and was sequenced on Illumina NovaSeq 6000 using NovaSeq 6000 S1 PE Reagent Kit (300 cycles) with an average of 3 × 10^7^ reads per DNA sample.

### Metaproteomics by nanoLC-MS/MS

The metaproteome samples were processed and analyzed as previously described (40). The protein precipitates were dissolved in SDS-PAGE sample loading buffer, loaded on an SDS-gel and run for 10 min. The gel pieces were cut, washed, and incubated with 25 mM 1,4-dithiothreitol (in 20 mM ammonium bicarbonate) for 1 h and 100 mM iodoacetamide (in 20 mM ammonium bicarbonate) for 30 min. The gel pieces were further destained, dehydrated, and proteolytically cleaved overnight at 37°C with trypsin (Promega, Germany). The peptide lysates were extracted and desalted using ZipTip-C18 tips (Merck Millipore, Germany). Afterward, the peptide lysates were re-suspended in 0.1% formic acid and injected to a nanoliquid chromatography mass spectrometry (nanoLC-MS/MS). Thus, the mass spectrometric analyses of peptide lysates were performed on a Q Exactive HF mass spectrometer (Thermo Fisher Scientific, USA) coupled with a TriVersa NanoMate (Advion, Ltd., UK) source in LC chip coupling mode. LC gradient, ionization mode, and mass spectrometry mode have been used as described before (51).

Data resulting from nanoLC-MS/MS analysis were analyzed using the Proteome Discoverer v.2.4 (Thermo Fisher Scientific, USA) using the SEQUEST HT algorithm. As a FASTA database, the protein-coding sequences of the metagenome (*n* = 164,604 sequences) were used. Search settings were set to trypsin (Full), max. missed cleavage: 2, precursor mass tolerance: 10 ppm, fragment mass tolerance: 0.02 Da. The false discovery rates (FDRs) were determined with the node Percolator (52) embedded in Proteome Discoverer and the FDR threshold was set at a peptide level of <1%. The same threshold was set for the protein FDR.

### Ple extraction, detection, and purification

The synthetic gene constructs were manufactured, codon optimized, and cloned into the pCold II by BioCat GmbH, Germany. Cells of *Escherichia coli* Origami (DE3) (Merck, Germany) were chemically transformed with pCold II vectors containing *ple-tan1*, *ple-tan2,* and *ple-solo*. The transformants were plated on Lysogeny Broth (LB) agar with 100 µg/mL of ampicillin plates.

Expression of the Ple-tan1, Ple-tan2, and Ple-solo was carried out by transferring the 2 mL pre-cultures to 500 mL Terrific Broth medium supplied with 50 µg/mL of ampicillin in 2 L baffled flasks. The cultures were incubated at 37°C, 120 rpm until an OD of 0.8–1.0 was reached. The cultures were cooled down on ice for 15 min and induced with 0.5 mM IPTG. Induced cultures were then incubated at 16°C, at 120 rpm in a MaxQ 4000 orbital shaker (Fisher Scientific, Germany) overnight.

Induced cultures were harvested the next day in an Avanti J-26 XPI centrifuge with a JA-14 rotor (Beckman Coulter, USA) at 6,000 × *g*, 4°C for 5 min. The pellets were frozen at −20°C for 20 min and resuspended in 50 mL of a Wash/Load buffer (50 mM NaH_2_PO_4_ × 2 H_2_O, 300 mM NaCl, pH 8.0) and 1 µL of Pierce Universal Nuclease for Cell Lysis (Thermo Fisher Scientific, USA). Cells were placed on ice and disrupted with a Sonopuls HD2070 sonicator366 (Bandelin, Germany) at 50% amplitude for 2.5 min two times with a 1 min break. Lysates were centrifuged at 14,000 × *g*, 4°C for 30 min in an Avanti J-26 XPI centrifuge with a JA-14 rotor (Beckman Coulter, USA) and filter sterilized with a 0.2 µM filter.

Pooled supernatants were further purified on a 5 mL cOmplete His-Tag Purification Column (Sigma-Aldrich, USA) connected to an ÄKTA Start Purification System (Cytiva, Germany). For protein elution, an elution buffer consisting of 50 mM NaH_2_PO4 × 2 H_2_O, 300 mM NaCl and 250 mM Imidazole at pH 8.0 with a 1 mL/min flow rate was used. The elution buffer was used in a gradient between 5% and 100%. The Ples were eluted at 12% of the gradient. About 3 mL of Ple-tan1, 1 mL of Ple-tan2 and Ple-solo were eluted.

About 10 µL of the fractions was loaded into a 12% SDS-PAGE gel and ran for 1 h at 25 V/cm in a Mini-PROTEAN Tetra Cell (Bio-Rad Laboratories, USA). The presence of Ples was detected by comparing the bands to a protein size standard (Precision Plus Protein Unstained Standards, Bio-Rad Laboratories, USA). Pure fractions were further pooled together, buffer exchanged with PBS and concentrated on a Pierce Protein Concentrator PES 10K MWCO (Thermo Fisher Scientific, USA) according to the manufacturer’s instructions.

The Ples that presented contamination by other proteins were further purified by size exclusion chromatography on a HiPrep 16/60 Sephacryl S-200 HR column (Cytiva, Germany) connected to an ÄKTA Start Purification System (Cytiva, Germany) in a buffer consisting of 25 mM Tris–HCl and 150 mM NaCl, pH 7.5. The Ples tan1 and solo which presented contamination by other proteins were further purified by size exclusion. Previous to the purification by size exclusion, Ple-tan1 and Ple-solo were concentrated and buffer was exchanged after His-Tag purification by using a Pierce Protein Concentrator PES 10K MWCO (Thermo Fisher Scientific, USA) according to the manufacturer’s instructions. After size exclusion purification, the proteins were further concentrated by using a Pierce Protein Concentrator PES 10K MWCO (Thermo Fisher Scientific, USA). The concentrations of the purified proteins were analyzed by using Qubit Protein Assay kits and quantified on a Qubit 3.0 Fluorometer (Invitrogen, CA, USA).

### Determination of enzyme optimum temperatures

The activities of Ple-tan1, Ple-tan2, and Ple-solo were tested at 20, 30, 40, and 50°C by measuring the formation of BT and T after degradation of ecovio FT. For the reaction, 10 µg of each Ple was incubated with 5 mg of ecovio FT in 1.5 mL Eppendorf tubes containing 500 µL of 100 mM sodium phosphate buffer, pH 7.4. Incubations were carried out at 300 rpm in an Eppendorf Thermomixer C (Eppendorf, Germany) for 48 h. Two sampling points of 50 µL each were taken, after 24 and 48 h of incubation. The reactions were carried out in triplicates. Three negatives per temperature containing only the buffer and ecovio FT were also included in the assay.

BT and T were detected with a 1260 Infinity II LC System (Agilent Technologies, USA) and separated through an Agilent Poroshell 120 HPH-C18 column (Agilent Technologies, USA) as described before (46). The separation was performed with a gradient of acetonitrile 99.9% HPLC grade (Fisher Scientific, USA) and 0.1% (vol/vol) formic acid (98–100% Suprapur, Sigma-Aldrich, USA) in Milli-Q water. The flow rate was set at 0.2 mL/min. About 1 µL of the sample was injected. Acetonitrile was increased from 5% to 44% until minute 12 and then to 70% at minute 15 remaining constant for another 3 min. BT and T formation was detected at 240 nm after elution times of 16.3 and 7.5 min, respectively.

### Ples enzymatic assays on ecovio FT, PCL, PBS, and pNPB

The activities of Ple-tan1, Ple-tan2, and Ple-solo were additionally analyzed with ecovio FT, PCL, and PBS as the substrate. For the reaction, 0.02 µg/µL of enzyme was incubated with 5 mg of either ecovio FT, PCL, and PBS in 500 µL of ammonium acetate buffer (40 mM), pH 7.4. The reactions were incubated at the Ples optimum temperature, 20°C, at 300 rpm in an Eppendorf Thermomixer C (Eppendorf, Germany). Incubations lasted 48 h and two sampling points were taken, after 24 and 48 h. Reactions without the enzymes were used as negative controls. Each assay was performed in triplicate. Product formation was qualitatively analyzed by either HPLC (ecovio FT reactions) as described previously or LC-MS (PCL and PBS reactions).

The ecovio FT reactions were then transferred to standard HPLC vials. BT, BHBT, and T were detected as described previously (46). The HPLC-MS analysis of the PBS and PCL degradation products was made as previously described (46).

*Km* and *kcat* were determined for Ple-tan1, Ple-tan2, and Ple-solo by measuring 4-nitrophenol after the hydrolysis of pNPB. For the reaction, different concentrations of pNPB (0.1–2.4 mM) were mixed with 0.005 µg/µL of enzyme in 60 µL of PBS, pH 7.4. Incubations were carried out in 96-well plates and at room temperature in a TECAN Infinite M200 plate reader (TECAN, Switzerland). Measurements were performed every 5 min for 1 h, at 405 nm and results were retrieved using the TECAN i-control v.1.5.14.0 software (TECAN, Switzerland). Standard curves were prepared by measuring the different concentrations of 4-nitrophenol dissolved in PBS, pH 7.4 buffer, and incubated under the same conditions as the enzymatic assays. Reactions without the enzymes were used as negative controls. Each assay was performed in triplicate. *Km*, *Vmax*, and *kcat* were determined by nonlinear least squares regression (http://biomodel.uah.es/en/metab/enzimas/MM-regresion.htm, last accessed 04.04.2022).

### Data analysis

#### Processing and assembly of metagenome sequence data

Raw read metagenome sequences were quality filtered and trimmed using Trimmomatic v0.39 (53), followed by removal of PhiX sequencing control reads using BBMap v38.90 (https://jgi.doe.gov/data-and-tools/software-tools/bbtools/). The quality-checked paired-end reads were then error corrected using the “Tadpole.sh” workflow implemented in BBMAP with the options “cc = t rollback = t pincer = t tail = t prefilter = t prealloc = t mode = correct.” The quality of the sequenced reads in all these steps was assessed using FASTQC (54). Finally, the high-quality reads were independently assembled using metaSPAdes v3.15.2 (55) with preset metagenomic options and a Kmer range of 21–127. Contigs longer than 500 bp were retained for downstream analyses.

#### Genome-resolved metagenomic binning

The resulting size-filtered metagenomic contigs were mapped against the corresponding error-corrected metagenomic reads using BBMap v38.90 (https://github.com/BioInfoTools/BBMap) with default settings and the “pairedonly = t” option. The mean base coverage per contig was used for unsupervised metagenome-resolved genome binning using MetaBAT2 v2.12.1⁠ (56) with the default settings and a minimum contig size of 1.5 kbp. The quality of MAGs was assessed using CheckM v1.1.3 and v2.1.0 wwith the “lineage_wf” command. MAGs were taxonomically assigned with GTDB-Tk v1.3.0 (57) ususing the “classify_wf” command with default settings and GTDB database version 95 (accessed 23 July2020) (58). The MAGs were dereplicated using dREP v3.2.2 (59) with default settings, resulting in a total of 35 representative MAGs for downstream analyses.

The abundance of these 35 MAGs was determined by mapping quality-filtered paired-end metagenomes. Genome-wide mapping was performed using CoverM v0.6.1 (https://github.com/wwood/CoverM, last accessed 24.11.2022) against a concatenated nucleotide sequence of the 15 representative genomes, requiring a read length of 75% and an identity threshold of 95%. Abundance values were calculated by dividing the mean coverage of each MAG by the total coverage of all MAGs, followed by multiplying this proportion by the total number of mapped reads. Final relative abundance values were calculated as a proportion of the sum of the calculated abundances in a sample.

#### Generation of gene sequence catalog and abundance estimation

Putative protein-coding gene sequences were predicted from the filtered contigs using Program v2.6.3 (60) in the metagenomic mode (option: -p meta), retaining only genes that were predicted to have been complete, with defined codon boundaries (i.e., categorized as “partial = 00”). This yielded a total of 1,485,086 complete non-redundant genes.

The protein-coding gene sequences from all metagenomes were clustered into a non-redundant set of genes with mmseqs2 v4.6.1 (61) as described in Duarte et al. (62) based on the “easy-linclust” workflow, applying default settings and the following options: global sequence identity (--min-seq-id 0.95) and sequence length overlap of the shorter sequence (-c 0.80). Following this procedure, we obtained a total of 178,655 complete non-redundant genes.

The abundance of these genes in the separate metagenomes was determined based on mapping the gene catalog separately against the high-quality metagenomes with BBMap v.V38.18. BBmap was performed using the following parameters: nodisk = t rpkm = $fpkm ambig = toss idfilter = 0.95 tossbrokenreads. This resulted in a common metric of normalized abundance of reads per kilobase million mapped reads (RPKM).

### Differential gene abundance analyses

The analysis of differential gene abundances was performed based on number of reads mapped to genes corresponding to MAGs using DESeq2 (63). Only genes with a log2 fold change with a *P*adj ≤ 0.05 were considered. Comparisons were made between polymers between attached and free-living fractions of the replicate samples.

### Metaproteome analysis

Metaproteomic quantification was performed by grouping the redundant proteins in protein groups with the strict parsimony principle. Those protein groups that explain at least one unique identified peptide were reported. The peptides that were not shared between different proteins or protein groups were used for the protein quantification. Quantification of peptides was performed with the Top3 approach as part of the Proteome Discoverer v.1.4 (Fisher Scientific, USA) software. The resulting data were then log2-transformed and normalized. Normalization was carried out by dividing the log2-transformed values by the median of the sample and multiplying by the mean of the entire data set as described before (64, 65). To determine protein synthesis in the film-attached and free-living fractions, we calculated fold changes of normalized values by subtracting the means of normalized film-attached triplicates to those of normalized free-living triplicates. Proteins synthesized only in the film-attached fractions, or presenting a fold change of ≥1, were considered to be upregulated proteins. Contrary, proteins synthesized only in the free-living fractions or presenting a fold change of ≤−1, were considered to be downregulated proteins. The *P* values were calculated from two-tailed *t* test. Values with *P* values ≤0.05 were considered significant. Calculations were carried out by using Microsoft Excel 2010 v.14.0.7252.5000.

The replicate consistency was determined by analyzing the distribution of the normalized areas (Fig. S10). Clustering of the normalized areas to each condition (ecovio FT, PBS, or PCL) was calculated and visualized with ComplexHeatmap v.2.1.0 (66). Venn diagrams were produced by using the online tool InteractiVenn (67). KO identifiers assigned to each gene after metagenomics were used to assign a KEGG pathway to each up-regulated or downregulated protein. Pathways were collected and total count of proteins per pathway was performed and the results were visualized. All R packages were used with R v.3.6.0 (68). All tools were run with default parameters.

The phylogenetic tree of the bins was constructed using the online UBCG pipeline (69) and visualized with iTOL v.6 (70).

## Data Availability

The nucleotide sequences of ple-tan1, ple-tan2, and ple-solo have been deposited to GenBank under the identifiers OP554724, OP554725, and OP554726, respectively. The mass spectrometry proteomics data have been deposited to the ProteomeXchange Consortium via the PRIDE (71) partner repository with the dataset identifier PXD038098. The metagenome reads were submitted to NCBI under the BioProject IDs PRJNA885412 and PRJNA887682.

## SUPPLEMENTAL MATERIAL

Supplemental file 1 aem.01060-23-s0001.pdf Fig. S1 to S10 and Tables S1 to S3.

ASM does not own the copyrights to Supplemental Material that may be linked to, or accessed through, an article. The authors have granted ASM a non-exclusive, world-wide license to publish the Supplemental Material files. Please contact the corresponding author directly for reuse.
