# Supplementary material for: Comparative biodegradation analysis of three compostable polyesters by a marine microbial community
Source: Appl Environ Microbiol. 2023 Nov 28;89(12):e01060-23. doi: 10.1128/aem.01060-23 (PMC10734441; doi:10.1128/aem.01060-23)
Supplement: Supplemental file 1 — Fig. S1 to S10 and Tables S1 to S3. [file aem.01060-23-s0001.pdf]

Comparative biodegradation analysis of three compostable polyesters by a marine microbial community

Supplementary information, includes: Supplementary figures 1-10, Supplementary tables 1-3

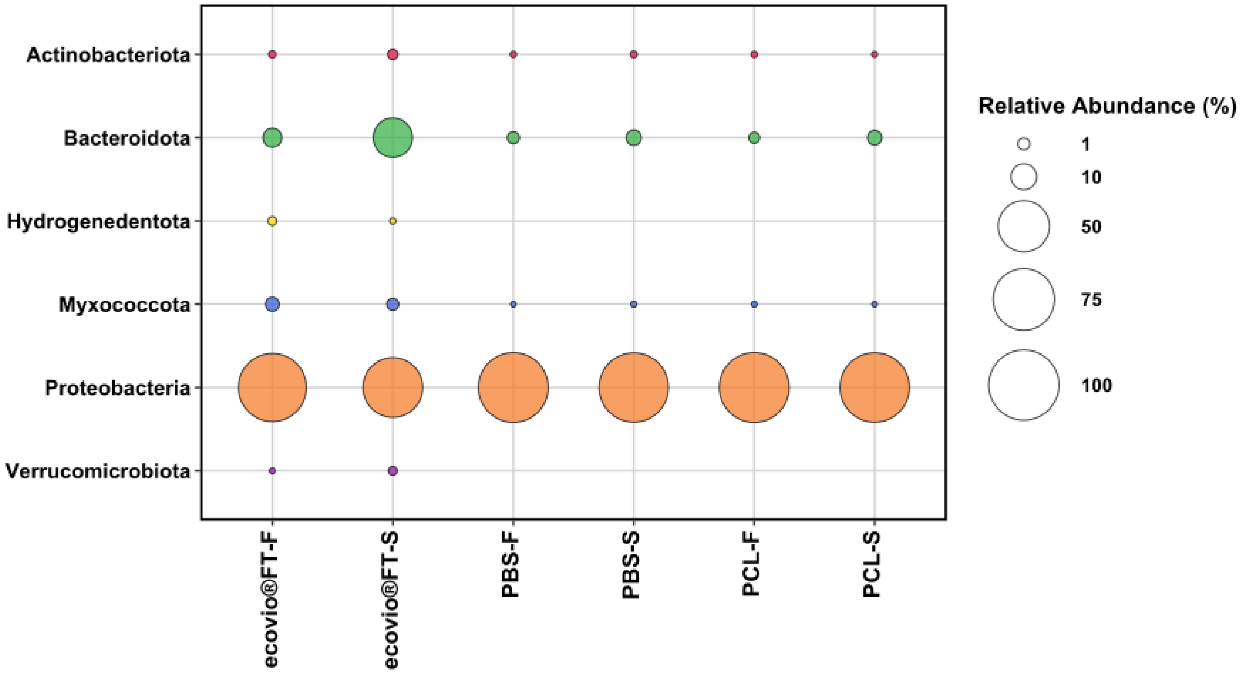

Figure S1: Taxonomic distribution of the microbial community at phylum level. The size of the bubbles represents percent abundance.

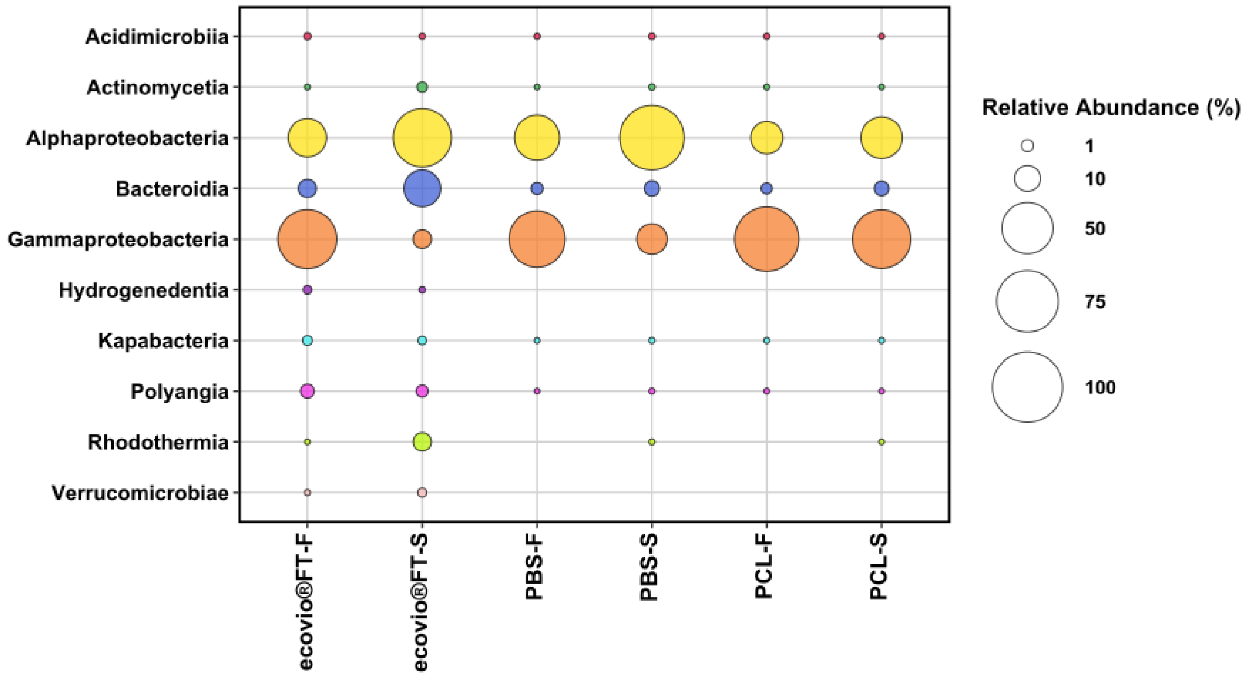

Figure S2: Taxonomic distribution of the microbial community at class level. The size of the bubbles represents percent abundance.

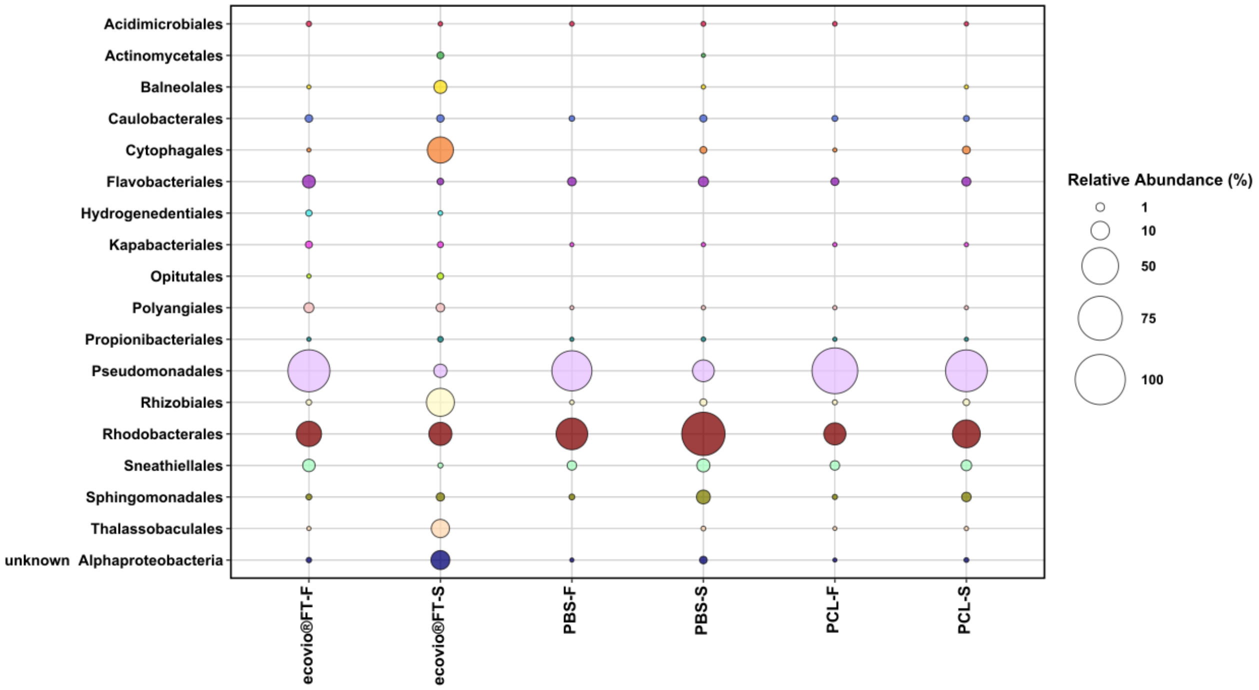

Figure S3: Taxonomic distribution of the microbial community at order level. The size of the bubbles represents percent abundance.

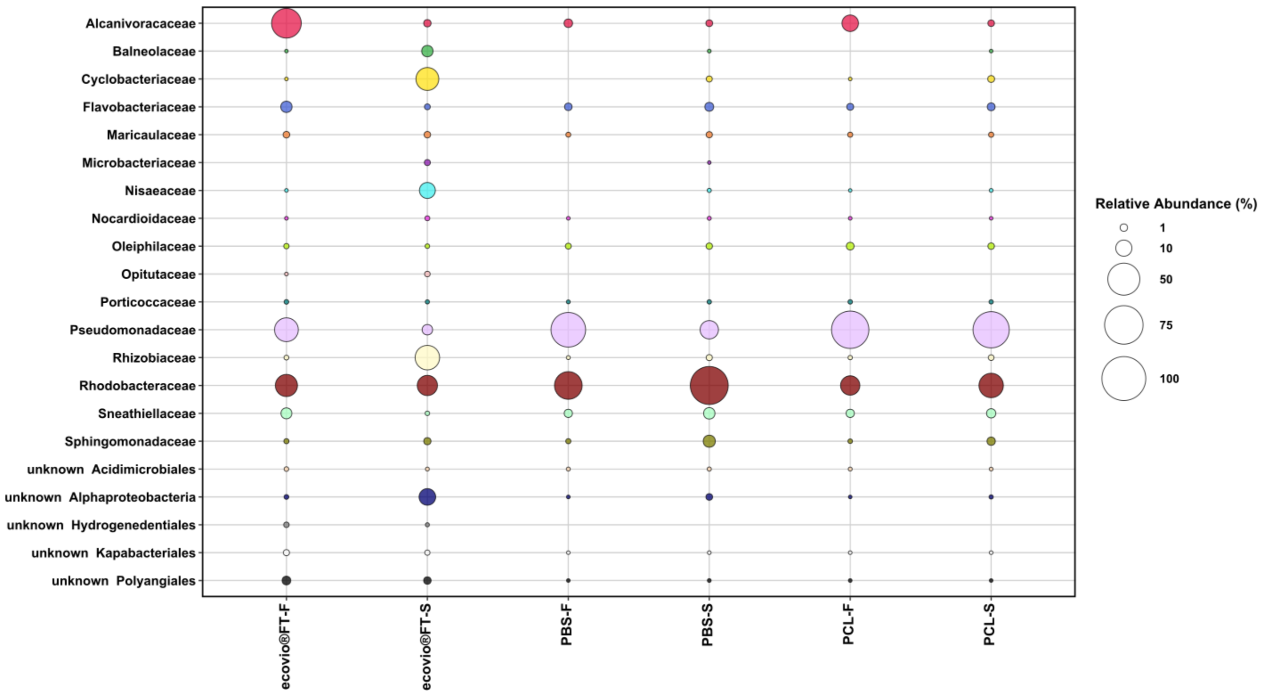

Figure S4: Taxonomic distribution of the microbial community at family level. The size of the bubbles represents percent abundance.

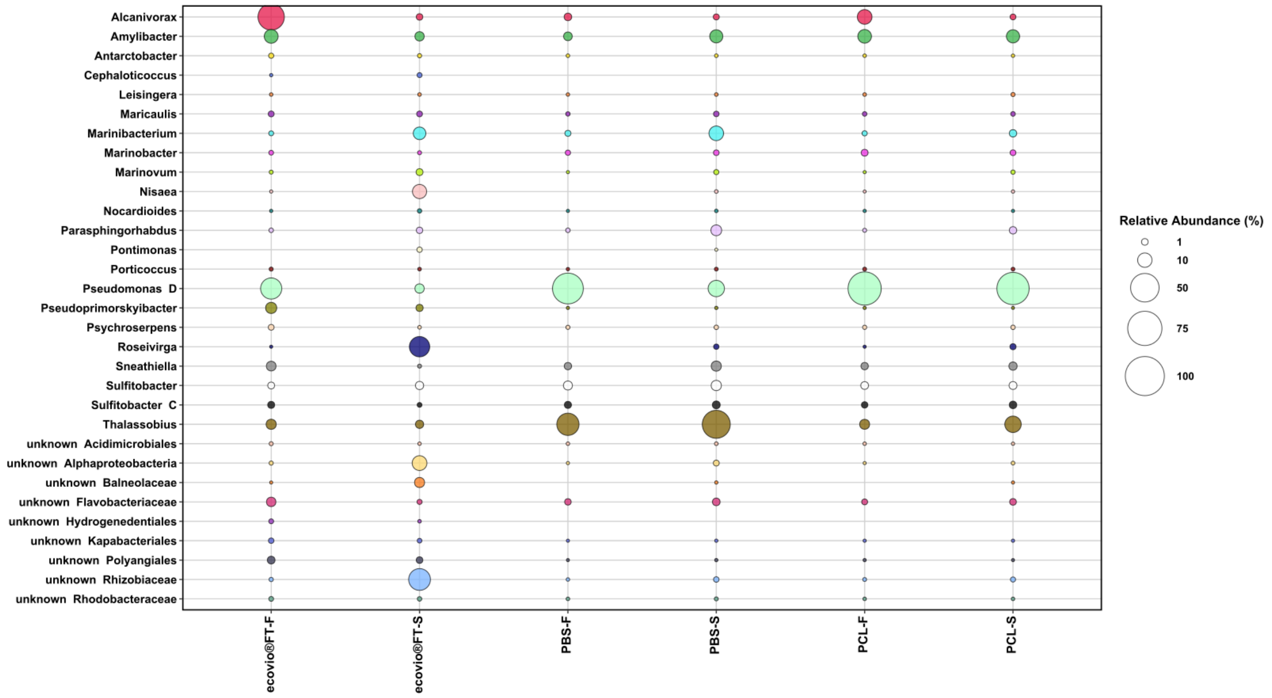

Figure S5: Taxonomic distribution of the microbial community at genus level. The size of the bubbles represents percent abundance.

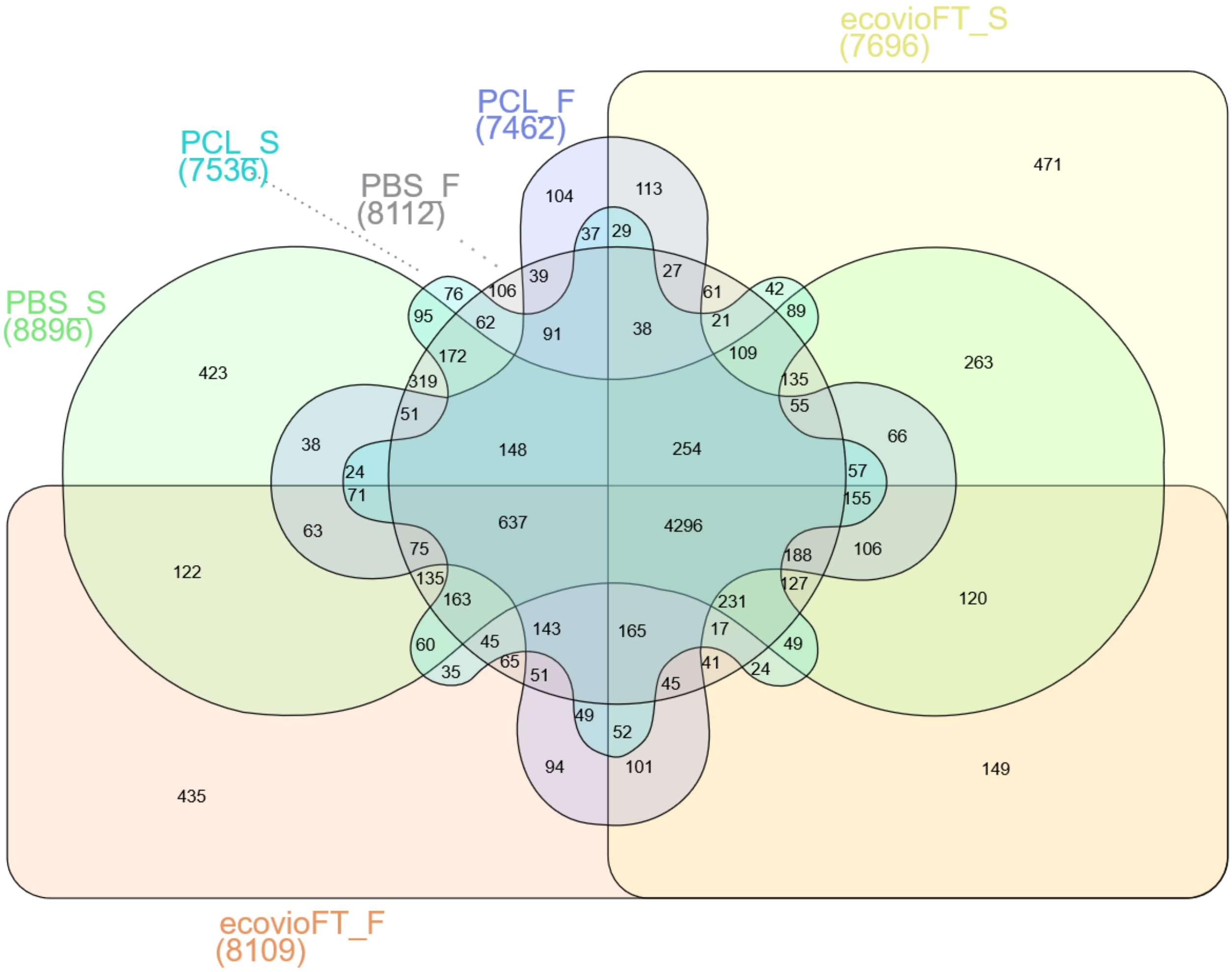

Figure S6: Venn Diagram of proteins formed under different experimental conditions.

A

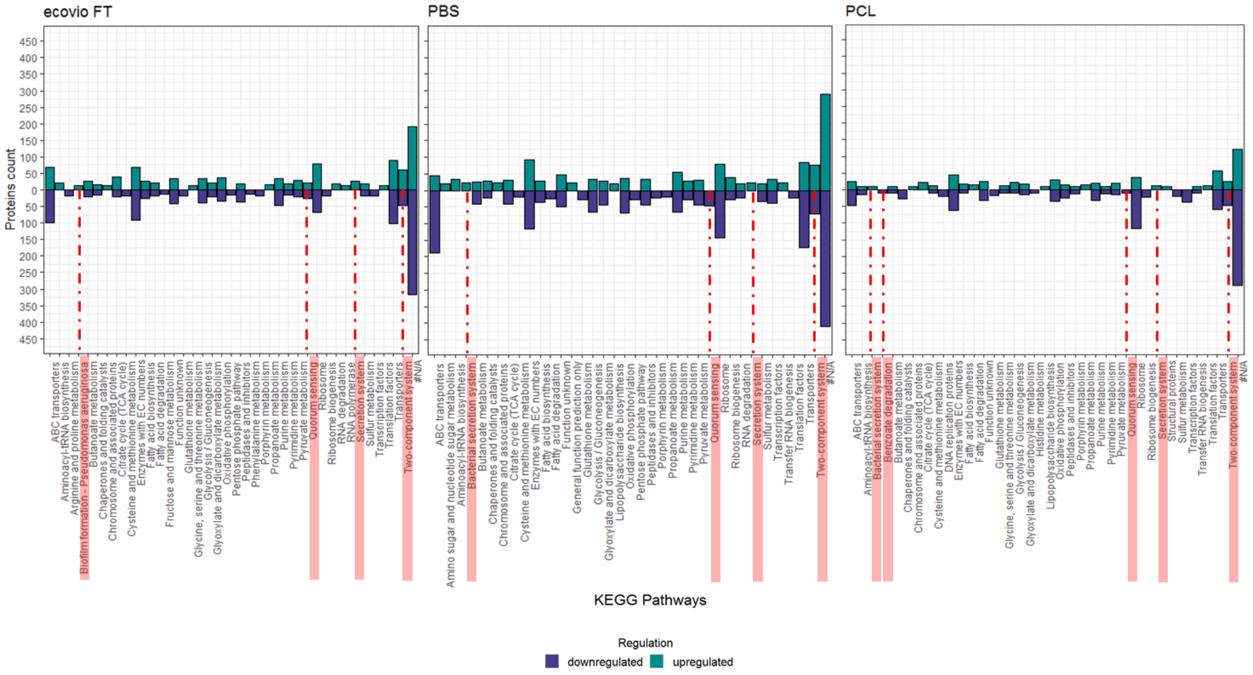

B

PATHWAY	UPREGULATED			DOWNREGULATED		
	ecovio®FT	PBS	PCL	ecovio®FT	PBS	PCL
Aminobenzoate degradation	3	2		2	5	2
Bacterial motility proteins	1	2				
Bacterial secretion system	11	23	10	6	9	2
Benzoate degradation	7	11	1	13	14	11
Biofilm formation - Escherichia coli	9	14	2	3	11	5
Biofilm formation - Pseudomonas aeruginosa	14	18	4	1	6	1
Biofilm formation - Vibrio cholerae	4	6	6	4	5	4
Caprolactam degradation	1					
Cell motility	4	2	1		1	
Chloroalkane and chloroalkene degradation		1	1	2	3	1
Flagellar assembly	4	13	7	2	2	7
Fluorobenzoate degradation	1	2	3	4	2	3
Methane metabolism	7	10	4	5	10	2
Naphthalene degradation			1		1	
Nitrotoluene degradation		2	1			1
Polycyclic aromatic hydrocarbon degradation	3	3	2	1	2	1
Prokaryotic defense system	6	11	1	1	2	4
Quorum sensing	20	10	9	27	46	11
Secretion system	25	24	12	3	7	9
Starch and sucrose metabolism	3	1		1	2	2
Steroid degradation	1	2		1		
Two-component system	60	77	25	46	71	48

Figure S7: Regulation of selected pathways under different experimental conditions. A) Regulation of metabolic pathways based on absolute numbers of up-and-downregulated proteins. B) Zoomed-in view of the regulation of selected pathways potentially related to biofilm formation and biodegradation.

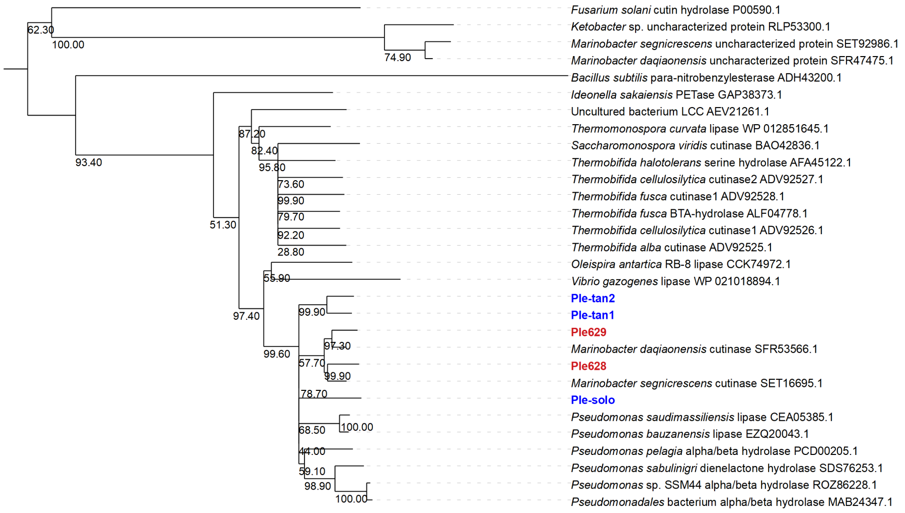

Figure S8: Phylogeny of the Ples identified in this study and their relatives. The numbers on the branches represent the bootstrap values. The Neighbor-joining tree was calculated by with Geneious® with 1,000 bootstrap replicates. The protein alignment was made with Clustal Omega.

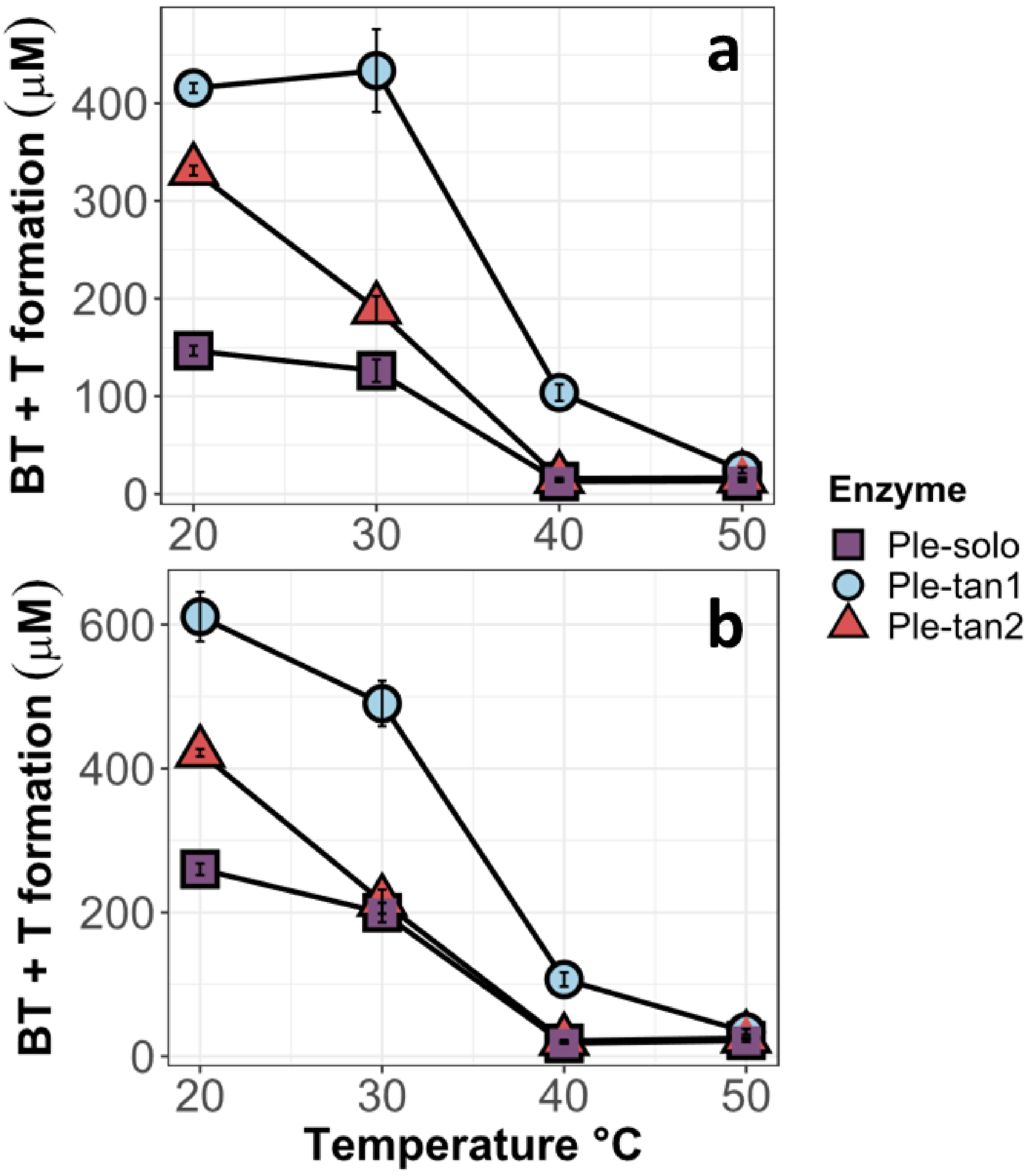

Figure S9: Temperature optima of Ple-tan1, Ple-tan2, and Ple-solo. Temperature optima of Ple-tan1, Ple-tan2, and Ple-solo. The formation of BT and T (in µM) was quantified after degradation of ecovio® FT at different temperatures by Ple-tan1 (blue circle), Pletan2 (red triangle) and Ple-solo (purple square). Two time points were taken, after 24 (a) and 48 h (b).

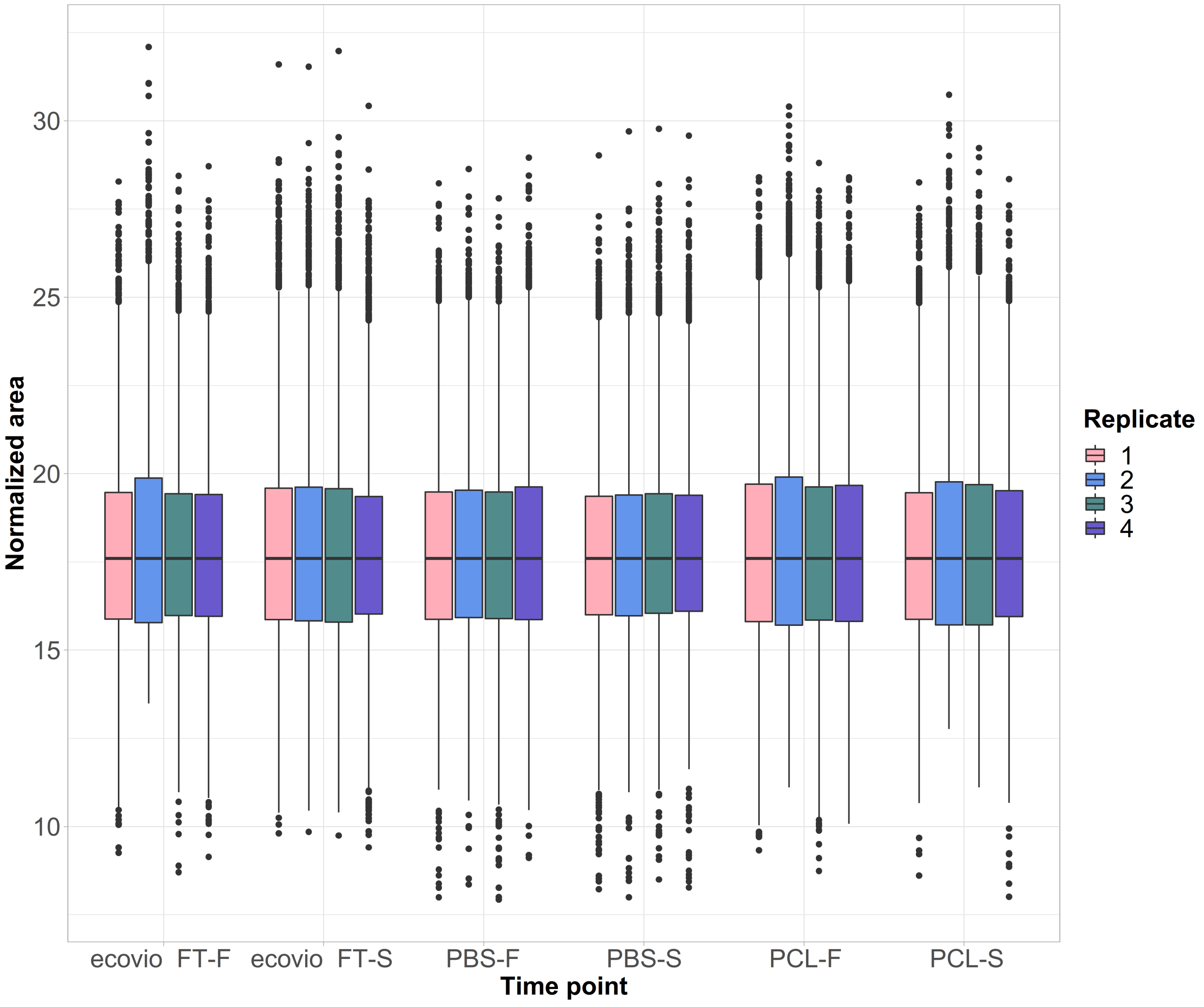

Figure S10: Comparison of the biological replicates for the metaproteome. Box plots show the 1.5 × interquartile range, the maximum and minimum data point within the range of samples represented by whiskers, the median value of the samples represented by the center line and the outliers of the different data sets (n = 4).

Table S1: Genome stats of MAGs.

**Study ID name**	MAG_ID	Completeness	Contamination	Strain_heterogeneity	MAG_Ids	#Contigs	TotalLength_
Acidimicrobiales_bin 25	EC_MBin.25	78.48	3.37	28.57	EC_MBin.25.fa	1307	3896747
Pontimonas_bin 27	EC_MBin.27	89.31	3.12	100	EC_MBin.27.fa	105	1450298
Nocardioides_bin 7	EC_MBin.7	90.54	1.56	0	EC_MBin.7.fa	586	3707529
Roseivirga_PB_bin 40	PB_MBin.40	98.9	0.3	0	PB_MBin.40.fa	14	4023689
Flavobacteriaceae_bin 22	EC_MBin.22	97.21	0.19	0	EC_MBin.22.fa	9	2952099
Psychroserpens_bin 41	EC_MBin.41	99.67	0.33	0	EC_MBin.41.fa	12	3403337
Kapabacteriales_bin 42	EC_MBin.42	96.07	0	0	EC_MBin.42.fa	18	3081701
Balneolaceae_bin 52	EC_MBin.52	99.72	0.94	0	EC_MBin.52.fa	23	3759651
Hydrogenedentiales_bin 53	EC_MBin.53	98.9	1.28	0	EC_MBin.53.fa	102	7443872
Polyangiales_bin 16	EC_MBin.16	95.81	2.87	0	EC_MBin.16.fa	105	7933502
Maricaulis_bin 40	EC_MBin.40	99.46	0	0	EC_MBin.40.fa	12	3146192
Rhizobiaceae_bin 21	PC_MBin.21	75.86	0	0	PC_MBin.21.fa	21	5599007
Rhodobacteraceae_bin 3	EC_MBin.3	99	2.15	15.79	EC_MBin.3.fa	204	6548356
Amylibacter_bin 29	PB_MBin.29	100	0.61	0	PB_MBin.29.fa	7	4448939
Amylibacter_bin 43	EC_MBin.43	96.28	0.76	0	EC_MBin.43.fa	115	4324215
Antarctobacter_bin 6	EC_MBin.6	99.15	0.27	0	EC_MBin.6.fa	18	4972749
Leisingera_bin 33	PC_MBin.33	97.26	3.35	30.43	PC_MBin.33.fa	386	4727832
Marinibacterium_bin 35	EC_MBin.35	98.08	0.53	0	EC_MBin.35.fa	29	5077940
Marinovum_PB_bin 42	PB_MBin.42	99.19	1.28	0	PB_MBin.42.fa	68	4778466
Pseudoprimorskyibacter_bin 44	EC_MBin.44	97.94	0.6	25	EC_MBin.44.fa	15	4125293

**Study ID name**	MAG_ID	Completeness	Contamination	Strain_heterogeneity	MAG_Ids	#Contigs	TotalLength_
Sulfitobacter_ bin 9	PB_MBin.9	99.36	0.48	0	PB_MBin.9.fa	26	4291996
Sulfitobacter_ bin 38	EC_MBin.38	77.34	2.14	0	EC_MBin.38.fa	727	2789054
Sulfitobacter_ PC_ bin 7	PC_MBin.7	98.08	1.12	75	PC_MBin.7.fa	25	3321653
Thalassobius_ bin 47	EC_MBin.47	99.36	0.32	50	EC_MBin.47.fa	16	3849804
Thalassobius_ PB_ bin 16	PB_MBin.16	97.76	1.18	0	PB_MBin.16.fa	73	3305869
Sneathiella_ bin 17	PC_MBin.17	100	1.3	33.33	PC_MBin.17.fa	4	4012830
Parasphingorhabdus_ PB_ bin 30	PB_MBin.30	99.09	1.51	0	PB_MBin.30.fa	7	3560352
Nisaea_ bin 30	EC_MBin.30	100	0.43	0	EC_MBin.30.fa	8	4540858
Alphaproteobacteria_bin 49	EC_MBin.49	95.7	0	0	EC_MBin.49.fa	28	1335152
Alcanivorax_ bin 24	EC_MBin.24	100	0.37	0	EC_MBin.24.fa	8	3741446
Marinobacter_ PB_ bin 43	PB_MBin.43	98.15	0.22	0	PB_MBin.43.fa	33	3413678
Marinobacter_ bin 48	PC_MBin.48	97.83	0.37	33.33	PC_MBin.48.fa	35	3480127
Porticoccus_ bin 19	PC_MBin.19	89.02	1.85	0	PC_MBin.19.fa	292	2238034
Pseudomonas_ bin 31	EC_MBin.31	96.12	0.95	0	EC_MBin.31.fa	17	3949044
Cephaloticoccus_ bin 50	EC_MBin.50	99.32	0.68	0	EC_MBin.50.fa	25	3912542

Table S2: Input data for Figure 3

	GENES						PROTEINS			
	F			S			F			
**Class**	**% in ecovio FT**	**% in PBS**	**% PCL**	**% in ecovio FT**	**% in PBS**	**% PCL**	**% in ecovio FT**	**% in PBS**	**% PCL**	**% in ecovio FT**
Alphaproteobacteria	3.09	0.37	0.28	0.19	0.09	0.08	0.51	0.28	0.49	0.39
Alphaproteobacteria	0.38	3.93	2.23	0.06	0.05	0.05	0.90	0.42	0.79	0.28
Actinomycetia	0.00	0.00	0.00	1.42	0.00	0.00	0.00	0.00	0.00	0.00
Alphaproteobacteria	1.11	7.39	3.01	5.82	0.87	0.47	1.28	1.31	4.44	8.00
Alphaproteobacteria	13.04	2.06	0.03	0.09	0.01	0.03	1.85	0.42	0.39	1.17
Alphaproteobacteria	0.80	0.16	0.56	0.12	0.22	0.10	0.51	0.28	0.39	0.61
Polyangia	0.00	0.01	0.25	0.04	0.03	0.00	1.15	0.70	0.89	0.11
Alphaproteobacteria	16.38	0.00	0.00	0.00	0.01	0.00	2.75	0.23	0.99	0.67
Gammaproteobacteria	0.01	0.00	0.39	0.00	0.02	0.00	0.06	0.05	0.10	0.00
Alphaproteobacteria	0.01	0.02	0.11	12.24	17.88	14.42	0.38	4.06	6.12	20.62
Bacteroidota	11.54	0.10	0.00	0.00	0.00	0.01	1.15	0.23	0.49	0.17
Gammaproteobacteria	14.81	40.79	68.89	0.00	0.00	0.00	22.31	5.60	23.10	0.56
Acidomicrobiia	0.09	0.05	0.06	0.00	0.00	0.00	0.00	0.05	0.00	0.00
Actinomycetia	0.00	0.00	0.00	2.92	0.00	0.00	0.00	0.05	0.10	0.06
Alphaproteobacteria	0.05	0.00	0.11	1.14	1.65	0.01	10.23	0.70	5.63	0.61
Alphaproteobacteria	0.00	0.00	0.00	7.97	13.71	15.70	0.13	0.09	0.30	0.94
Alphaproteobacteria	0.00	0.02	0.00	9.61	2.87	0.10	0.38	1.63	1.28	5.61

Table S2 continued from previous page

				**GENES**					**PROTEINS**	
Gammaproteobacteria	15.60	43.67	0.00	0.00	0.00	0.00	32.61	62.72	25.27	4.61
Alphaproteobacteria	0.02	0.13	0.03	0.15	0.19	0.46	0.00	0.00	0.10	0.00
Alphaproteobacteria	0.01	0.05	0.00	11.07	18.50	19.64	0.58	0.42	1.97	13.56
Alphaproteobacteria	0.32	0.15	0.06	0.07	0.05	0.09	0.06	0.05	0.10	0.28
Bacteroidota	0.01	0.02	0.00	8.37	12.95	16.20	0.19	1.91	1.88	6.45
Alphaproteobacteria	0.01	0.05	0.33	4.24	0.40	0.00	0.26	0.14	0.20	0.06
Bacteroidota	11.24	0.15	0.00	0.00	0.00	0.01	0.13	0.09	0.30	0.06
Gammaproteobacteria	0.01	0.02	0.00	10.25	15.66	14.40	0.00	0.65	1.48	2.39
Kapabacteria	0.01	0.02	0.03	0.02	0.01	0.00	0.13	0.09	0.10	0.17
Gammaproteobacteria	0.19	0.58	20.59	0.00	0.00	0.00	0.45	0.37	0.30	0.56
Alphaproteobacteria	0.01	0.02	0.03	7.61	10.13	0.02	2.75	0.37	2.57	0.61
Alphaproteobacteria	0.17	0.09	0.00	0.01	0.02	0.00	3.96	1.12	1.97	0.33
Alphaproteobacteria	0.00	0.01	0.00	0.01	0.00	16.92	7.93	5.60	3.36	6.00
Gammaproteobacteria	0.04	0.09	2.99	0.04	0.00	0.00	0.32	0.33	0.30	0.17
Alphaproteobacteria	0.00	0.01	0.00	2.93	4.64	1.29	0.00	0.14	0.49	1.61
Verrucomicrobiae	0.00	0.00	0.00	6.06	0.00	0.00	0.06	0.05	0.00	0.06
Rhodotermia	0.00	0.01	0.00	7.51	0.04	0.00	0.38	0.33	0.39	1.78
Hydrogenedentia	11.05	0.01	0.03	0.00	0.00	0.00	0.06	0.09	0.30	0.06
	0.00	0.00	0.00	0.00	0.00	0.00	6.52	9.43	13.43	21.46
	100.00	100.00	100.00	100.00	100.00	100.00	100.00	100.00	100.00	100.00

Table S3: Input data for Figure 4

	MG			MP				
Protein ID	ecovio FT	PBS	PCL	ecovio FT	PBS	PCL	Specie	Bins
Hyd467	0	0	0	-15	-15	0	n.d.	n.d.
Est056	-3.59576	-1.93713	0	0	-15	0	*Amylibacter*	bin 43
Lip861	1.633848	3.116871	0	0	2.56	-3.84	*Pseudomonas*	bin 31
Hyd905	4.077675	2.399802	5.010139	0	1.79	0	*Alcanivorax*	bin 24
Hyd026	4.308193	2.305671	4.763072	0	0	15	*Alcanivorax*	bin 24
Hyd450	4.106315	2.252433	0	3.59	0	0	*Alcanivorax*	bin 24
Lip174	4.286087	2.468077	5.275697	0	-4.4	-2.48	*Alcanivorax*	bin 24
Lip810	4.14515	2.630224	0	0	0	15	*Alcanivorax*	bin 24
Hyd934	4.02641	2.293335	4.753048	0	1.27	0	*Alcanivorax*	bin 24
Hyd936	4.443065	2.561871	5.047177	2.75	0	0	*Alcanivorax*	bin 24
Hyd195	-3.65035	0	1.051203	0	-1.55	0	*Sulfitobacter*	PC_bin 7
Hyd027	1.623252	3.212674	0	0	0	1.44	*Pseudomonas*	bin 31
Est479	0	0	0	-15	-2.46	-15	n.d.	n.d.
Hyd688	4.463478	2.623525	0	4.95	0	3.02	*Alcanivorax*	bin 24
Hyd355	-6.06256	-2.71334	-1.09262	0	-2.64	0	*Marinibacterium*	bin 35
Hyd360	1.831057	3.455552	0	0	2.31	0	*Pseudomonas*	bin 31
Hyd151	-5.91814	-2.8199	-1.72404	-15	0	0	*Marinibacterium*	bin 35
Lip103	4.274469	2.382363	0	0	0	3.97	*Alcanivorax*	bin 24
EstB137	1.928034	0	0	3.34	0	0	*Sulfitobacter*	bin 9
Hyd226	-6.21135	-2.49845	-1.57432	-3.06	-2.07	0	*Marinibacterium*	bin 35
Hyd603	-6.43279	-2.70112	-1.5225	-15	0	0	*Marinibacterium*	bin 35
Hyd634	0	0	0	0	-2.33	0	n.d.	n.d.
Hyd566	1.723432	3.326404	0	2.78	1.81	1.33	*Pseudomonas*	bin 31
EstB745	0	0	0	15	0	1.39	*Amylibacter*	bin 29
Hyd842	-3.79861	0	0	0	0	2.36	*Sulfitobacter*	PC_bin 7
Hyd900	1.609566	3.15083	0	0	1.34	0	*Pseudomonas*	bin 31
Hyd994	-14.4397	-6.0219	-6.26244	0	0	15	*Roseivirga*	PB_bin 40
Hyd037	-9.23638	-5.0425	-2.21159	-15	0	0	Rhizobiaceae	bin 21
Hyd519	-13.9798	-5.3678	-6.60507	0	0	-15	*Roseivirga*	PB_bin 40
Hyd253	1.656443	3.280567	0	0	-1.66	0	*Pseudomonas*	bin 31

		MG			MP			
Lip186	-15.4734	-6.78297	-8.40244	-5.83	0	0	*Roseivirga*	PB_bin 40
Hyd958	1.781202	3.21259	0	0	1.22	0	*Pseudomonas*	bin 31
Hyd986	4.11002	2.15211	0	-2.07	0	0	*Alcanivorax*	bin 24
Ple-solo	1.720762	3.364631	0	9.99	5.48	6.99	*Pseudomonas*	bin 31
Hyd066	1.587635	3.220406	0	0	15	0	*Pseudomonas*	bin 31
Hyd916	-9.63378	-3.53663	-1.90118	-4	0	0	Rhizobiaceae	bin 21
Ple-tan1	1.824961	3.537869	0	3.58	3.03	0	*Pseudomonas*	bin 31
Ple-tan2	1.711153	3.464858	0	7.33	4.16	3.63	*Pseudomonas*	bin 31
Hyd031	0	0	0	-3.72	-3.1	0	n.d.	n.d.
Hyd205	1.717854	3.462332	0	0	3.24	2.09	*Pseudomonas*	bin 31
Hyd330	-8.95992	-3.40365	-2.38198	0	15	0	Rhizobiaceae	bin 21
Lip285	1.730592	3.428653	0	3.4	0	3.84	*Pseudomonas*	bin 31
Est530	0	0	0	0	0	-1.75	*Thalassobius*	PB_bin 16
Lip392	0	0	-1.44148	-15	0	-15	*Thalassobius*	bin 47
Hyd620	-8.52666	-1.90636	-2.63575	0	-2.05	0	Rhizobiaceae	bin 21
Est729	-9.11621	-3.19877	-1.88342	-5.23	0	1.75	Rhizobiaceae	bin 21
Hyd022	0	0	-1.56901	0	0	-15	*Thalassobius*	bin 47
Hyd162	1.561715	3.488448	0	0	2.47	0	*Pseudomonas*	bin 31
Hyd703	0	0	-1.44819	0	0	-15	*Thalassobius*	bin 47
Lip528	0	0	1.710142	0	2.9	0	*Marinobacter*	PB_bin 43
Hyd199	1.677197	3.387073	0	2.12	3.66	0	*Pseudomonas*	bin 31
Hyd507	1.676719	3.391498	0	0	15	0	*Pseudomonas*	bin 31
Est192	4.388763	2.304701	0	4.39	-15	0	*Alcanivorax*	bin 24
Hyd307	1.434085	3.40281	0	0	2.76	0	*Pseudomonas*	bin 31
Hyd663	1.642617	3.30779	0	0	3.2	0	*Pseudomonas*	bin 31
